# Melatonin targets ferroptosis through bimodal alteration of redox environment and cellular pathways in NAFLD model

**DOI:** 10.1042/BSR20230128

**Published:** 2023-10-06

**Authors:** Moumita Saha, Sanjib Das, Krishnendu Manna, Krishna Das Saha

**Affiliations:** 1 Cancer Biology and Inflammatory Disorder Division, CSIR-Indian Institute of Chemical Biology, Jadavpur, Kolkata 700032, India; 2 Department of Food and Nutrition, University of Kalyani, CSIR-Indian Institute of Chemical Biology, Jadavpur, Kolkata 700032, India

**Keywords:** Ferroptosis, Lipid peroxidation, Melatonin, NAFLD

## Abstract

Ferroptosis is a non-conventional cellular death caused by lipid peroxide induced iron deposition. Intracellular lipid accumulation followed by generation of lipid peroxides is an hallmark of non-alcoholic fatty liver disease (NAFLD). Melatonin (MLT) is an important pineal hormone with tremendous antioxidant and anti-inflammatory properties. Various studies targeted ferroptosis in different diseases using melatonin. However, none of them focused the intrinsic mechanism of MLT’s action to counteract ferroptosis in NAFLD. Hence, the present study investigated the role of MLT in improvement of NAFLD-induced ferroptosis.

HepG2 cells were treated with free fatty acids (FFAs) to induce *in vitro* NAFLD state and C57BL/6 mice were fed with high-fat diet (HFD) followed by MLT administration.

The results indicated that MLT administration caused the recovery from both FFA- and HFD-induced ferroptotic state via increasing GSH and SOD level, decreasing lipid reactive oxygen species (ROS) and malondialdehyde (MDA) level, increasing Nrf2 and HO-1 level to defend cells against an oxidative environment. MLT also altered the expression of two key proteins GPX4 and SLC7A11 back to their normal levels, which would otherwise cause ferroptosis. MLT also protected against histopathological damage of both liver tissue and HepG2 cells as depicted by Oil Red O, HE staining and immunofluorescence microscopy. MLT also had control over pAMPKα as well as PPARγ and PPARα responsible for lipid homeostasis and lipogenesis.

In brief, MLT exerted its multifaceted effect in FFA- and HFD-induced NAFLD by retrieving cellular oxidative environment, reducing lipogenesis and lipid peroxidation and modulating Nrf2/HO-1 and GPX4/SLC7A11 axis to combat ferroptosis.

## Introduction

Non-alcoholic fatty liver disease (NAFLD) is a chronic liver disorder induced by metabolic stress. NAFLD covers a variety of diseases from more simple forms like steatosis to more complex form cirrhosis and is the most prevalent liver disorder in the world [2]. Lipid peroxidation caused by oxidative stress is considered as a crucial factor in the pathogenesis of NAFLD. NAFLD is characterized by accumulation of lipid droplets in hepatocytes, hepatic cellular death, inflammatory cells infiltration and mostly fibrosis [3]. Non-alcoholic steatohepatitis (NASH) is an intermediate stage of NAFLD [4]. NASH is characterized by lipid droplet accumulation in hepatocytes, hepatic cell death, inflammatory cell infiltration, and, most importantly, fibrosis. While simple steatosis has a good prognosis, progression to NASH is a serious risk factor for cirrhosis and carcinogenesis [5,6]. Although number of theories about the pathogenesis of NASH have been proposed, hepatic cell death triggered by excess lipid accumulation is thought to be a likely cause of inflammation [7]. Several studies using genetically modified mouse models or chemical inhibitors have already revealed that apoptosis and necroptosis are associated with the pathophysiology of NASH [8]. Tsurusaki et.al. reported that hepatic ferroptosis is a crucial trigger for the onset of inflammation associated with NASH [9]. Metabolic disorder-related iron deposition increases the risk of hepatocyte expansion, related inflammation and fibrosis serving as a critical factor for NASH and therefore NAFLD [10].

Studies reported that chronic iron accumulation and dysregulated metabolic pathways plays an essential role by inducing lipid peroxidation, intracellular reactive oxygen species (ROS) generation, and mitochondrial dysfunction ultimately leading to ferroptosis in the liver [11,12]. Pieces of evidence suggest ferroptosis is also involved in the pathogenesis of several neuronal and kidney disorders besides cell death of certain types of tumor cells [13,14]. Although the detailed role of ferroptosis in liver disease is still not clearly understood. Ferroptosis is also characterized by disappeared mitochondrial cristae and increased mitochondrial membrane density without any significant changes in the nucleus [15]. Increased ROS accumulation causes depletion of reduced glutathione (GSH) and downregulates glutathione peroxidase 4 (GPX4) enzyme activity leading to ferroptosis [16]. Cystine-glutamate antiporter xCT (SLC7A11)-mediated cystine uptake is important for GSH synthesis which inhibits ferroptosis since GPX4 efficiently removes lipid peroxides from the cells [17].

Melatonin (N-acetyl-5-methoxytryptamine, MLT), primarily synthesized by the pineal gland, acts as a key regulator of the sleep–wake cycle and circadian rhythm [18]. Besides these, it also has a role in immunomodulation [19], bone metabolism [20], cardio-protection [21], neuroprotection [22] and metabolic regulation [23]. MLT controls the endogenous oxidative status of the cell by indirectly stimulating certain antioxidant enzymes such as GPX4 [24] and superoxide dismutase (SOD) [25,26]. Since ferroptosis is a type of non-apoptotic iron-dependent cell death associated with ROS [16]. Hence, we assumed that melatonin and ferroptosis are correlated, but the underlying mechanism should be further explored.

The Nrf2 (nuclear factor erythroid 2-related factor 2) signaling pathway is associated with detoxification and elimination of ROS [27]. Nrf2 plays an essential role in regulation of antioxidant genes which eventually exerts anti-inflammatory functions [28]. Nrf2 also regulates the expression of antioxidants by regulating the transcription of ARE (antioxidant responsive element)-dependent genes to balance cellular redox homeostasis [29]. Recent studies discovered that MLT reduced diabetes-induced kidney damage [30] and recovered neuronal damage [31] by activating the Nrf2/HO-1. Studies also reported that MLT protects against erastin [32] or RSL3-induced ferroptosis [33] in cancer cells.

SREBP-1 and PPARγ mediates the effects of nutrients and hormones on the expression of lipogenic genes [34]. Studies also reported that ghrelin-induced up-regulation of lipogenesis in hepatocytes is mediated by mTOR through its interaction with peroxisome proliferator-activated receptor (PPAR) [35]. In both primary rat hepatocytes and human hepatoma cell lines, the administration of oretic acid caused the activation of SREBP-1 while suppressing the phosphorylation of AMPK [36]. Studies also reported that, SREBP-1 is crucial for FAS response [37]. Melatonin showed its promising effect in reducing hepatic lipid buildup and dyslipidemia brought on by HFD in Syrian hamsters [38].

Different studies have explored the antioxidant nature of melatonin, the most important pineal hormone responsible for the coordination of circadian rhythm. Studies also reported the beneficial effect of melatonin administration against iron deposition-induced cell death i.e, ferroptosis, and its possible regulation Nrf2/HO-1 pathway to control osteoporosis [39], reprogramming of hepatic core clock oscillations [40], inhibited oxidative stress in cryopreserved ovarian tissues [41] and so on. However, no studies have yet been conducted to assess the beneficiary effect of melatonin in NAFLD-related ferroptosis. Hence, the present study primarily focused on the bimodal effect of melatonin to curb the ferroptotic cell death by regulating the proteins involved in both Nrf2/HO-1 and lipogenic axis with concomitant regulation of intracellular redox status in NAFLD model.

## Methodology

### Cell culture and steatosis induction in HepG2 cells with oleic and palmitic acids

Hepatoma cell lines have frequently been used as in vitro alternatives to primary human hepatocytes over the years because of their unlimited life span, stable phenotype, high availability, and easy handling. However, their major limitation is the lower expression of some metabolic activities compared with hepatocytes. Moreover, HepG2 is most used human hepatoma cell line for drug metabolism and hepatocytotoxicity studies due to its non-tumorigenic nature, structural homology to epithelial cells and high proliferation rates. Hence, in the present study HepG2 cells have been used to develop ferroptotic model [42].

The human hepatocellular carcinoma cell line (HepG2) was obtained from NCCS, Pune and grown in complete DMEM [10% (v/v) FBS and 1% PSN (Penicillin, Streptomycin, and Neomycin). The cells were kept humidified in an incubator set to % CO_2_ and 37°C temperature.

The cellular fatty liver/hepatic steatosis model was established using oleic acid (OA) and palmitic acid (PA). After dissolving the PA in ethanol, the OA/PA mixture was combined in a 2:1 ratio (2 mM) [1]. After that, the mixture was dissolved in BSA and vibrated overnight at 37°C. Before each experiment, the solution combination was freshly made. MLT was dissolved in DMEM growth media and administered to cells at various doses for subsequent studies.

### Animals and experimental protocol and non-alcoholic fatty liver disease model

Male wild-type C57BL/6 mice weighing 20–22 g were obtained from the CSIR-Indian Institute of Chemical Biology animal house. Before beginning the experimental procedures, the mice were kept in regular laboratory settings with access to food and water in a sterile environment for one week and allowed to adjust to their new surroundings. All studies were carried out in accordance with the Institutional Animal Care and Use Committee guidelines and were approved by the CSIR-Indian Institute of Chemical Biology’s Institutional Ethics Committee (IEC). All animal experiments took place at CSIR-Indian institute of chemical biology. All of the studies were carried out in the institutional animal house’s dissection room.

Thirty-two experimental mice were randomly assigned into four groups: control, high-fat diet (HFD), HFD + MLT D1 (10 mg/kg), and HFD+ MLT D2 (20 mg/kg). The mice were fed a HFD, and MLT (dissolved in 1× PBS) was injected intraperitoneally (i.p.) every alternate day for 2 months, whereas a control group of mice received an identical volume of 1× PBS and their body weight was measured weekly. At the end of experimental procedure, all of the animals were anaesthetized with an intraperitoneal injection of 100 mg/kg ketamine and 10 mg/kg xylazine hydrochloride before being killed via cervical displacement to collect blood serum and plasma for additional experimental investigation. Livers were cut out, weighed, and kept at −80°C for further experiments. For histological and immunofluorescence investigation, a piece of the liver was immediately fixed in neutral buffered saline (10% NBF). Establishment of *in vivo* and *in vitro* model and treatment regime is depicted in Supplementary Figure S1.

### Measurement of reactive oxygen species generation

Measurement of hydrogen peroxide generation can be used to quantify ROS [43]. Cells were then incubated with the DCF-DA (2′,7′-Dichlorofluorescin diacetate) for 25 min. After washing two times with 1× PBS, the fluorescence intensity was determined by FACScan flow cytometer (Becton Dickinson).

### Analysis of mitochondrial membrane potential using flowcytometry

Mitochondrial membrane potential (MMP) was analyzed using JC1 (1,1′,3,3′-tetraethyl-5,5′,6,6′-tetrachloroimidacarbocyanine iodide), a lipophilic dye with cationic nature. Approximately 10 μl of 200 μM JC-1 dye (2 μM in final concentration) was added to samples and kept for 20 min at 37°C. Cells were then washed and analyzed using flowcytometry (BD FACSAria™, BD BioSciences, San Jose, CA, U.S.A.).

### Oil Red O staining of cellular lipids

Oil Red O staining was used to detect accumulation of fatty acids (Wako, 154–02072). HepG2 cells were fixed with 4% formaldehyde at 37°C for 30 min. After that, cells were washed with 1× PBS. Oil Red O solution was added to the cells and incubated for 20 min, and washed with distilled H_2_O. Cells were observed in light microscope (Olympus IX).

### Determination of GSH and SOD activity

The GSH and SOD activity was measured using Reduced Glutathione (GSH) Assay Kit (Sigma Aldrich, U.S.A.: Cat.No. MAK364) and SOD Assay Kit (Sigma Aldrich, U.S.A.; Cat.No. 19160) based on the manufacturer’s instructions and measured using a Microplate Reader (Bio-Rad 680, U.S.A.).

### Analysis of MDA activity

MDA level is used to estimate lipid peroxidation. MDA level of cell lysates were measured using (Thermo Fisher Sci., U.S.A.) Lipid Hydroperoxide (LPO) Assay Kit as per the given instructions in the product manual.

### Estimation of cholesterol, triglycerides, and liver injury markers (AST and ALP)

Analysis of cholesterol, triglycerides and level of liver injury markers (AST and ALP) were analyzed using protocol provided with respective kits (Arkray Inc., Japan; Cat. No. 71LS200, 71LS100, 76MB101, 77MB101).

### Measurement of iron content in liver and HepG2 cell lysate

Iron concentration in both liver lysate and HepG2 Cell lysate were analyzed using kit (Iron Assay Kit; Colorimetric, ab83366, Abcam), according to the manufacturer’s instructions.

### BODIPY staining of lipid ROS

HepG2 cells were then fixed using 4% formaldehyde followed by washing with 1× PBS. This was followed by the addition of BODIPY to the culture dishes. Coverslips were then mounted on slides and observed under Confocal Microscope.

### MitoSoX red

HepG2 Cells were then fixed using 4% formaldehyde followed by washing with 1× PBS. This was followed by the addition of MitoSoX red to the culture dishes and incubation. Coverslips were then mounted on slides and observed under confocal microscope.

### Histopathological evaluation

The liver tissue was fixed by submerging it in NBF for 24 h. After being dehydrated with graded alcohol, the paraffin-embedded fixed tissue was sectioned with a microtome at a thickness of 4 μm. MT (Masson’s trichrome) and H&E (Hematoxylin and Eosin) staining were used to assess major pathological alterations.

### Immunofluorescence and confocal microscopy

Differentiated HepG2 cells, the cellular fatty liver/hepatic steatosis model, were established using oleic acid [OA] and palmitic acid [PA]). After dissolving the PA in ethanol, the OA/PA mixture was combined in a 2:1 ratio (2 mM). After that, the mixture was dissolved in BSA and vibrated overnight at 37°C. Before each experiment, the solution combination was freshly made. MLT was dissolved in DMEM growth media and administered to cells at various doses for subsequent studies) were fixed with 4% formalin followed by washing with 1× PBS. Permeabilization buffer was then used to permeabilize cells. The cells were then exposed to blocking buffer followed by treatment with primary antibody kept overnight at 4°C. Followed by cells on coverslips were exposed to fluorescent-dye conjugated secondary antibodies and cover slips were then mounted on slides and observed under confocal microscope (Leica SP8).

### Western blot analysis

Proteins from each sample was separated in SDS (10% dodecyl sulfate, sodium salt)-PAGE (polyacrylamide gel electrophoresis) and then transferred on to PVDF (polyvinylidene fluoride) membranes. Then, the membranes were blocked using blocking buffer (5% BSA/Tris Buffered saline Tween) for 1.5 h at room temperature (25–28°C), followed by incubation with primary antibodies at 4°C overnight. After incubation with secondary antibodies for 1–2 h at ambient room temperature, then the detection was done using NBT/BCIP chromogenic substrate and the relative expression of the respective proteins were analysed using ImageJ software (U.S.A.).

### siRNA transfection

NRF2 gene expression was knocked down by transfecting 1 × 10^6^ HepG2 cells with siRNA-NRF2 (sc-37030, Santa Cruz, U.S.A.) or siRNA control. Lipofectamine 3000 (Invitrogen, U.S.A.) was used to perform transient siRNA transfection, according to the manual of the manufacturer. After 24 h of transfection, HepG2 cells were administered with FFA and different concentrations of MLT. The efficiency of transfection was analysed using Western blotting.

### Statistical analysis

All data are represented as means ± SEM. Differences between three or more groups were compared with one-way ANOVA followed by post-hoc Tukey’s analysis. Differences between two groups were compared with a two-tailed *t*-test using Prism version 9 (GraphPad Software, San Diego, CA, U.S.A.). Statistically significant differences were considered when two-tailed *P-*value was < 0.05.

## Results

### NAFLD-linked ferroptosis was induced by FFA in HepG2 cells

**Figure 1:**
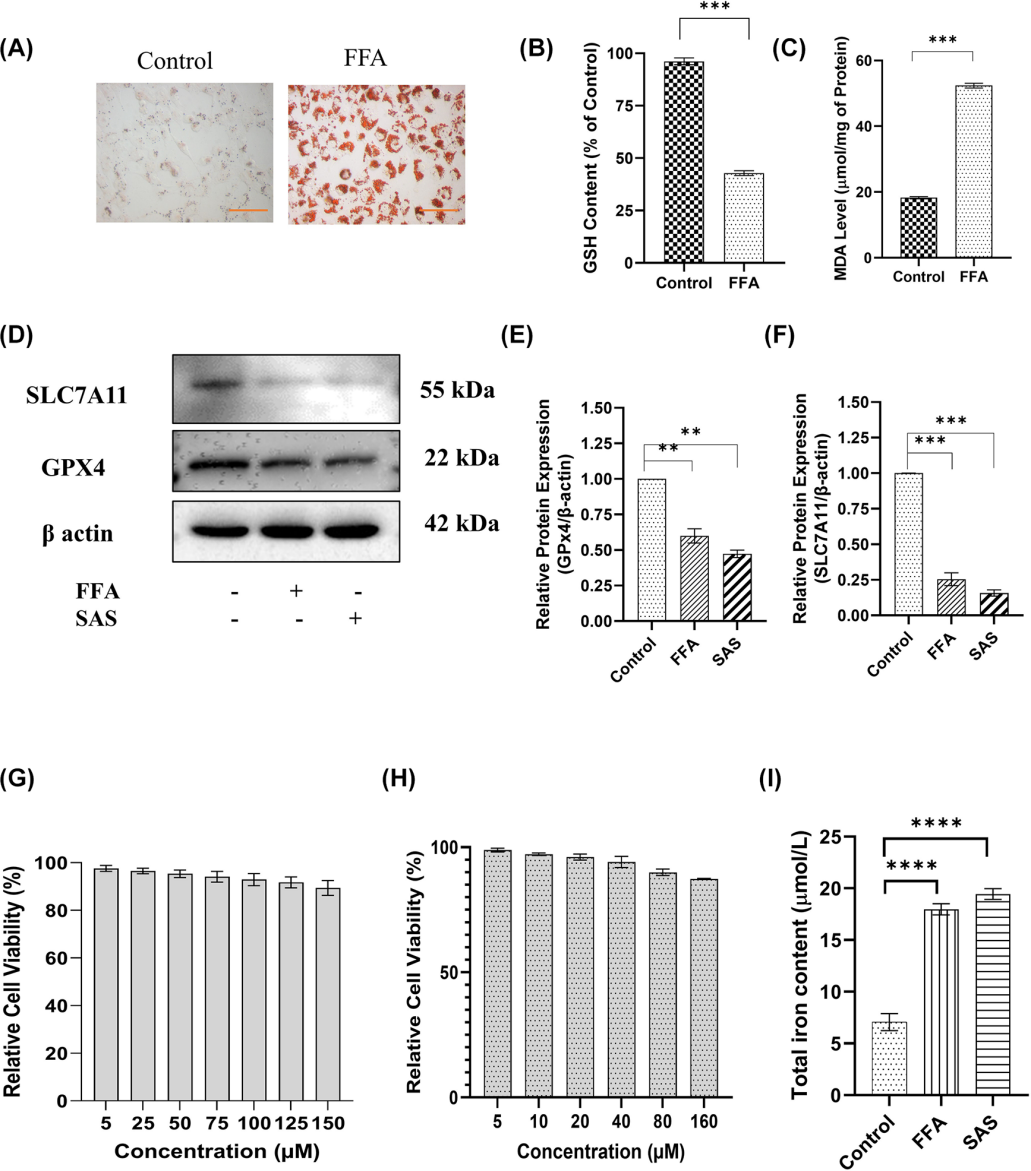
NAFLD-linked ferroptosis was induced by FFA in HepG2 cells (**A**) Oil Red O staining of lipid droplets in both control and FFA-treated cells. (**B**) Bar graph of GSH and (**C**) MDA content signifying changes in oxidative environment of cells. (**D**) Representative Western blot, and (**E,F**) bar graphs show the quantification of SLC7A11/β-Actin and GPX4/β-Actin, respectively. Panels (**G,H**) represent viability of HepG2 cells at different concentrations of MLT and SAS. Panel (**I**) represents total iron content in FFA/MLT-treated HepG2 cells. Data are represented as the mean percentage ± SEM (*n*=3); ****P<0.0001, ***P<0.001, **P<0.01, *P<0.05; ns, non-significant.

Ferroptosis is a form of regulated cell death characterized by accumulation of intracellular iron and enhanced lipid peroxidation due to impairment of cysteine glutathione–glutathione peroxidase 4 (SLC7A11-GPX 4) antioxidant defence. In the present study, primarily ferroptosis was induced by administration of FFA (PA+OA) in HepG2 cells. Accumulation of lipid droplets and changes in the intracellular oxidative environment is considered as important factors contributing to NAFLD which eventually leads to ferroptosis [44,45]. Oil red O staining of HepG2 cells indicated accumulation of lipid droplets upon FFA treatment (Figure 1A). Decreased levels of reduced glutathione were also noted in FFA-treated group signifying changes in the level of free radicals (Figure 1B). MDA level, being an important marker of lipid peroxidation, had also increased in FFA treated group compared with control (Figure 1C). Western blot analysis of key proteins involved in SLC7A11-GPX4 antioxidant defence axis were analyzed. The result revealed the decreased expression of SLC7A11 and GPX4 in FFA treated groups compared with control, a similar phenomenon as in treatment with SAS indicating possible initiation of ferroptosis (Figure 1D–F). Different studies showed the plausible effect of melatonin (MLT) against NAFLD due to its lower cytotoxicity (Figure 1G) and antioxidant potential [46,47]. Approximately 5, 25 and 50 µM concentrations of MLT had been used for future studies owing to its least cytotoxic potential. According to Yu et al., sulphasalazine (SAS) can act as an inducer of ferroptosis in breast tumor cells [48]. Observation of cell death revealed that treatment with SAS showed lower toxic effects in HepG2 cells (Figure 1H), and due to this it had been considered as a positive control for ferroptosis. Approximately 80 μM concentration of SAS had been chosen as an effective dose for further study.

The induction of the ferroptotic event was further confirmed by the level of intracellular iron (Figure 1I). Data indicated decrease in intracellular iron content with increasing doses of MLT. Collectively, it can be concluded that FFA was involved in the initiation of ferroptosis by lipid peroxidation and simultaneous changes in the endogenous oxidative environment as well as modulating intracellular iron deposition pattern in HepG2 cells.

### Melatonin improves overall effect of NAFLD-mediated ferroptosis

**Figure 2:**
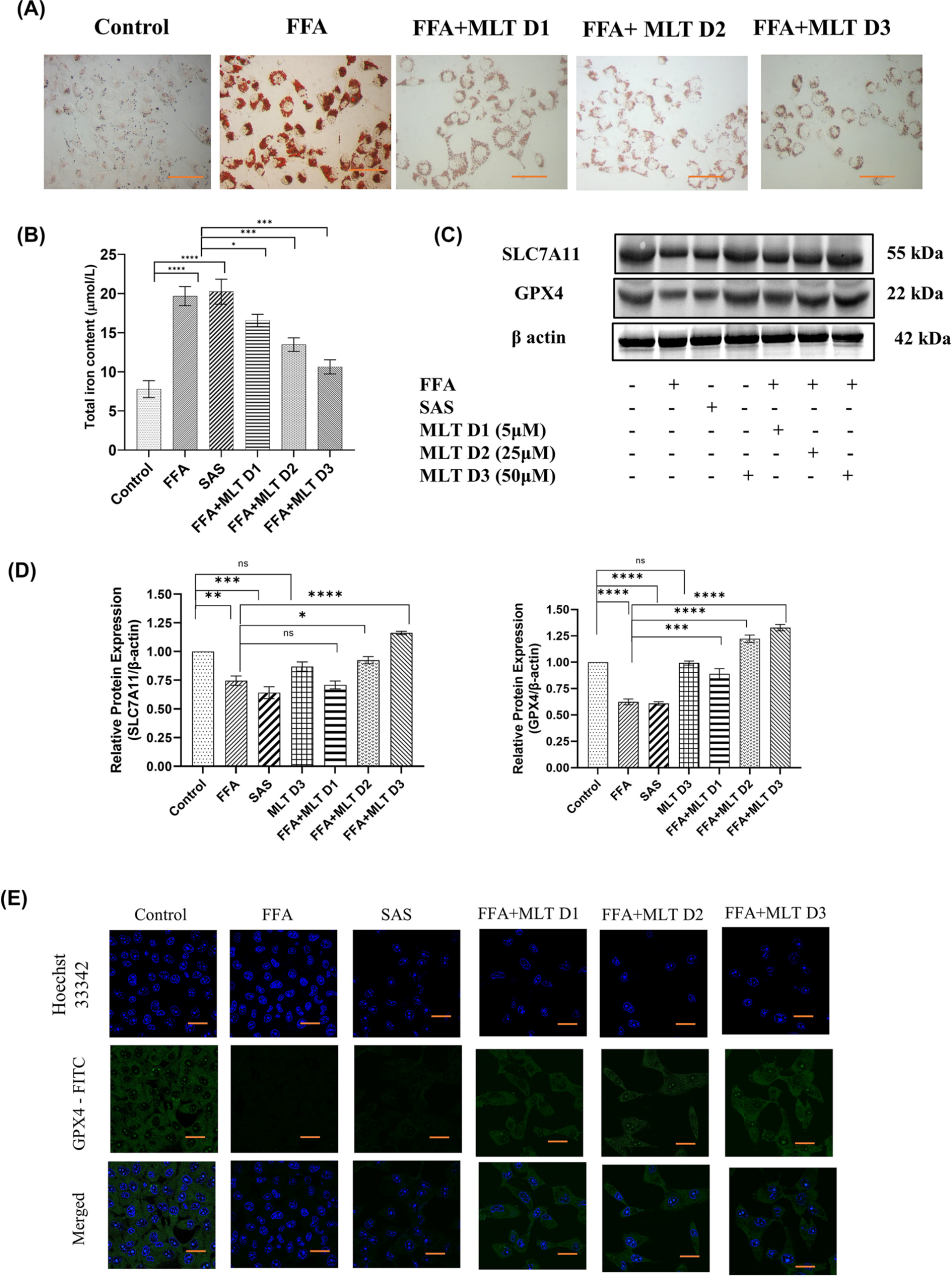
Melatonin improves overall effect of NAFLD-mediated ferroptosis Basic parameters indicating the effect of melatonin on NAFLD. (**A**) Oil Red O staning of lipid droplets in FAA- and MLT-treated groups. (**B**) Bar graph shows quantification of intracellular iron content. SAS used here as positive control. (**C**) Western blot of SLC7A11 and GPX4 in different groups of treated cells. (**D**) Bar graph shows the quantification of SLC7A11/β-Actin and GPX4/β-Actin. (**E**) The analysis of Expression of GPX4 using confocal microscopy. Data are represented as the mean percentage ± SEM (*n*=3); ****P<0.0001, ***P<0.001, **P<0.01, *P<0.05; ns, non-significant.

FFA-mediated lipid droplets accumulation is an important factor contributing to NAFLD. Oil Red O staining of HepG2 cells showed decreased lipid accumulation in MLT treated groups compared with FFA-treated group (Figure 2A). Sulphasalazine (SAS) had been used here as positive control and for further *in vitro* studies. Accumulation of intracellular iron from labile iron pool is the causative factor of ferroptosis [49]. MLT has shown a promising approach in this regard. Quantification of intracellular iron revealed a decrease in iron content of the cells treated with MLT in a dose-dependent manner. Cells treated with FFA+MLT D3 (50 µM) had shown significant decrease in intracellular iron compared with control and other doses of MLT (Figure 2B). Immunoblot analysis depicted a substantial increment of SLC7A11 and GPX4, upon MLT treatment. These two key proteins associated with glutathione synthesis and dysregulated during ferroptosis (Figure 2C,D). Figure 2E indicates confocal microscopic analysis of GPX4 expression, which supports the finding of western blot analysis of GPX4. These observations suggests that MLT played a protective role in ferroptosis.

### Melatonin recovers HepG2 cells from FFA-induced ferroptosis via modifying Nrf2/HO-1-mediated signaling

**Figure 3:**
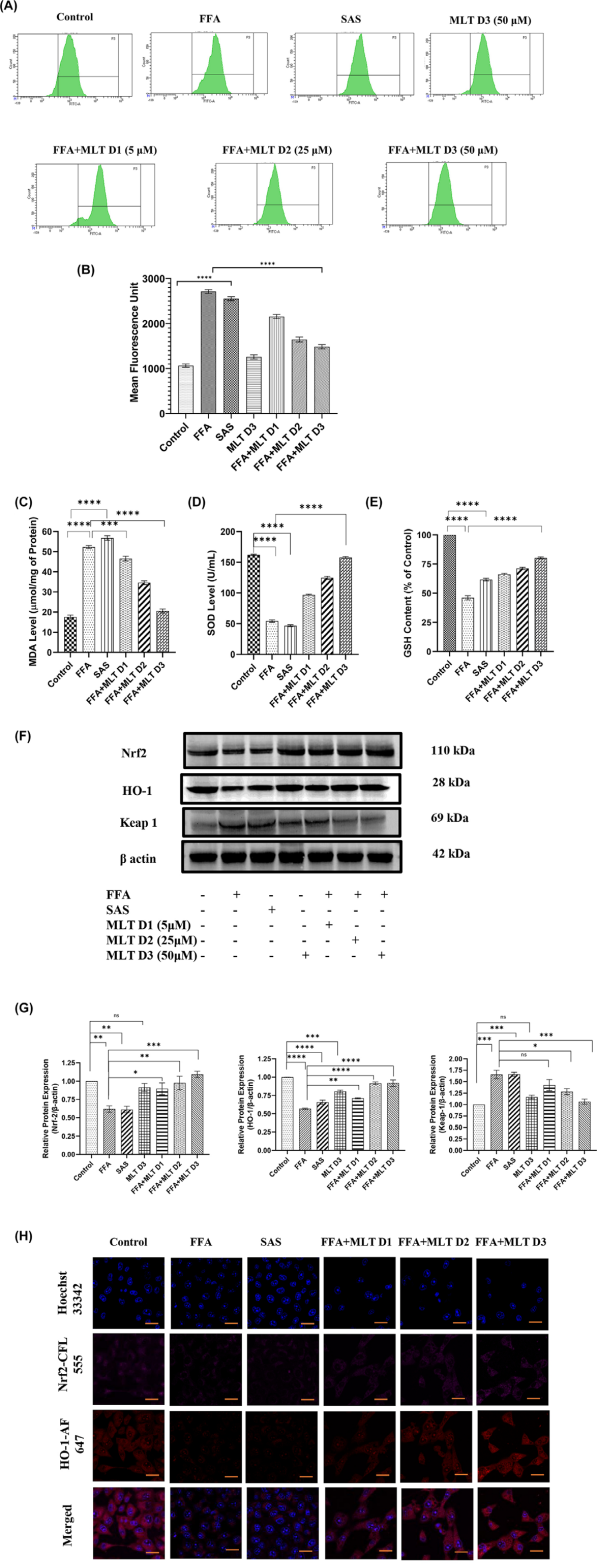
Melatonin recovers HepG2 cells from FFA-induced ferroptosis via modifying Nrf2/HO-1 mediated signaling Qualitative and quantitative analysis of intracellular oxidative status and involvement of Nrf2/HO-1 pathway in NAFLD-related ferroptosis. (**A**) Flow cytometric analysis of ROS generation in different treatment groups using DCFDA, and (**B**) bar graph represents mean fluorescence unit of ROS levels in different groups. Panels (**C–E**) represent MDA, SOD and GSH content in different treated groups. (**F**) Western blot analysis of Nrf2, HO-1 and Keap-1. (**G**) Bar graphs show the quantification of Nrf2/β-Actin, HO-1/β-Actin and Keap-1/β-Actin, respectively. (**H**) Representative immunofluorescence images shown Nrf2 and HO-1 expression in HepG2 cells depending on different treatments; scale bar: 50 µm. Data are represented as the mean percentage ± SEM (*n*=3); ****P<0.0001, ***P<0.001, **P<0.01, *P<0.05; ns, non-significant.

Increased lipid peroxidation in ferroptosis arises from an iron-dependent ROS accumulation [49]. The transcription factor Nrf2 is an important regulator of cellular antioxidant system, which controls the gene expression of proteins responsible for defence against electrophilic and oxidative stresses [50]. Analysis of intracellular ROS generation addressed the free radical scavenging potential of MLT. MLT at its higher dose (FFA+MLT D3) reduced the level of free radicals compared with only FFA- and SAS-treated cells (Figure 3A,B). MLT had also decreased MDA level indicating a reduction of lipid peroxidation (Figure 3C). MLT exhibited potent ROS scavenging activity [51]. Cells treated with MLT showed a dose-dependent increase in antioxidant enzyme SOD and consequent increase in GSH level (Figure3D,E). Nrf2 is an important regulator of redox homeostasis. Inhibition of Keap-1 and subsequent translocation of Nrf2 to nucleus causes transcription of antioxidant genes including HO-1 [52]. Western blot analysis of Nrf2, Keap-1 (an E3 ubiquitin ligase, act as inhibitory protein of Nrf2) and HO-1 demonstrated an increasing pattern of Nrf2 and HO-1 and decreasing pattern of Keap-1 with the increasing doses of MLT, suggesting the regulatory role of MLT in modulation of Nrf2/HO-1 axis for increasing level of antioxidants in FFA-treated cells and maintaining redox homeostasis (Figure 3F,G). Figure 3H depicts the confocal microscopy imaging for the expression of Nrf2 and HO-1 protein, which also supports the results of immunoblot analysis.

**Figure 4:**
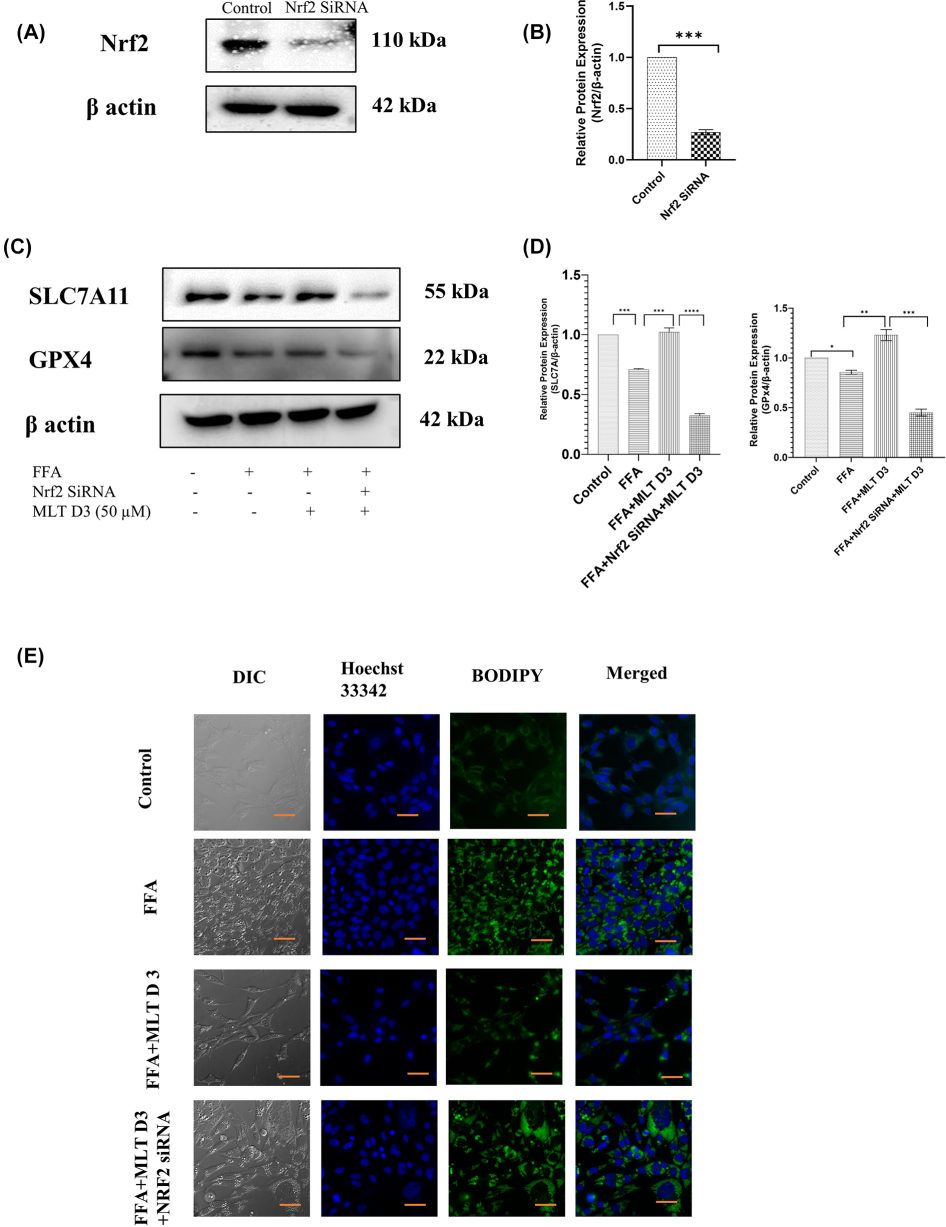
Melatonin recovers HepG2 cells from FFA-induced ferroptosis via modifying Nrf2/HO-1-mediated signaling SLC7A11 and GPX4 expression was verified using Nrf2 Si-RNA transfection. (**A**) Western blot analysis of Nrf2 in control and SiRNA-treated groups. (**B**) Bar graphs show the quantification of Nrf2/β-Actin. (**C**) Western blot analysis of SLC7A11 and GPX4. (**D**) Bar graphs show the quantification of SLC7A11/β-Actin and GPX4/β-Actin, respectively. (**E**) Immunofluorescence images of HepG2 cells of different treated groups using BODIPY; scale bar: 50 µm. Data are represented as the mean percentage ± SEM (*n*=3); ****P<0.0001, ***P<0.001, **P<0.01, *P<0.05; ns, non-significant.

To further investigate the involvement of the Nrf2 pathway in ferroptosis induced by FFA, Nrf2-siRNA was used to knockdown the expression of Nrf2. This was followed by Western blotting to confirm significant reduction in Nrf2 expression after transfection (Figure 4A,B). SLC7A11 and GPX4 are two important proteins involved in induction of ferroptosis. Western blot analysis revealed up-regulation of these proteins in FFA+MLT D3 group and their down-regulation in FFA+MLT D3+Nrf2 SiRNA group (Figure 4C,D) suggesting Nrf2 as a key mediator of ferroptosis. Figure 4E consists of BODIPY staining of lipid droplets in HepG2 cells, which indicates increased deposition of lipid droplets in FFA+MLT D3+Nrf2 SiRNA group further validating the role of Nrf2 in this regard. Therefore, cumulatively all these results imply MLT to be a potent antioxidant having control over Nrf2-mediated signaling.

### Melatonin controlled mitochondrial microenvironmental changes during ferroptosis

**Figure 5:**
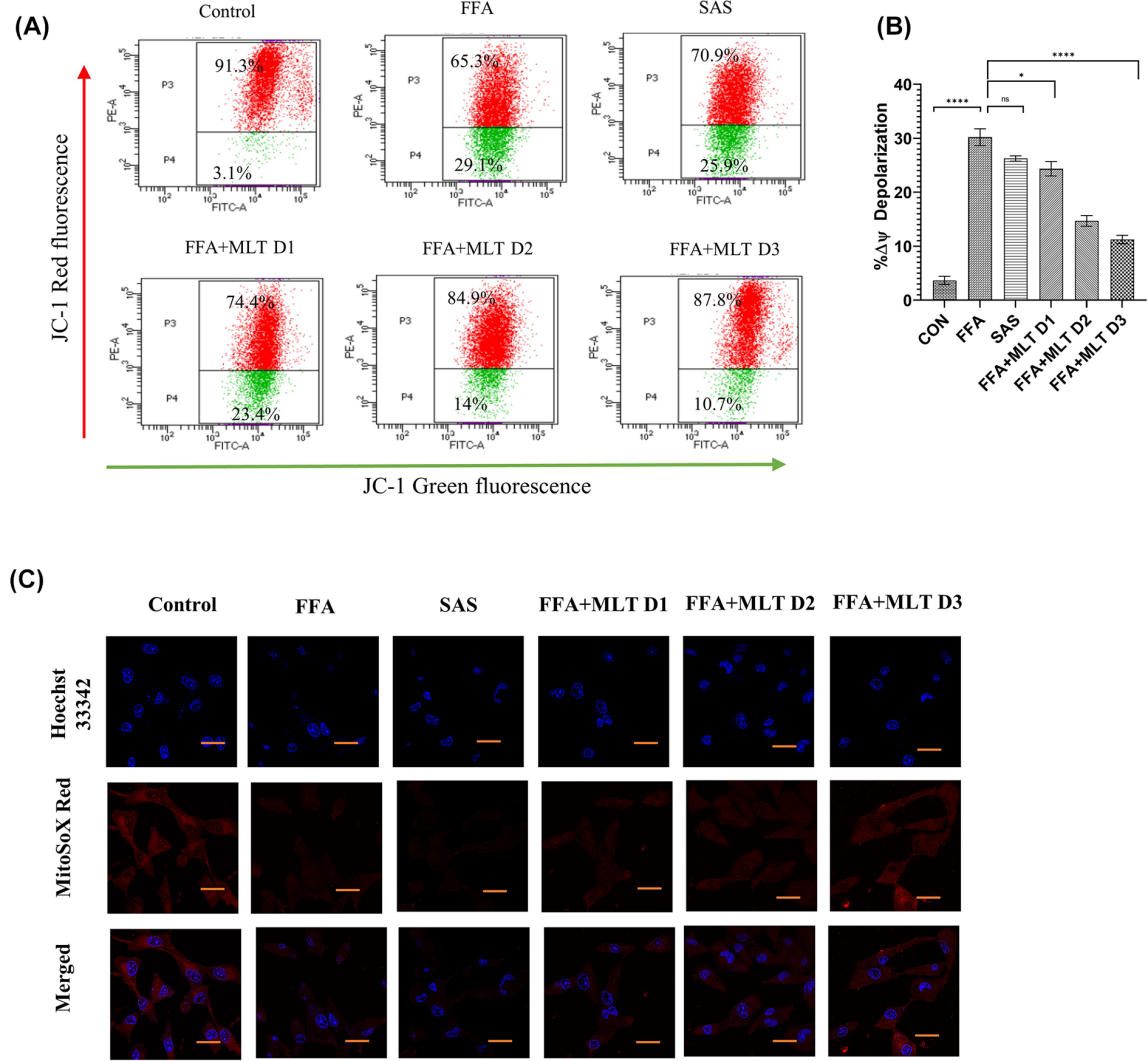
Melatonin controlled mitochondrial environmental changes during ferroptosis: changes in mitochondrial membrane potential and mitoROS (**A**) Flow cytometric analysis of mitochondrial membrane potential using JC1 in different treatment groups. (**B**) Bar graph represents percentage changes of depolarization of mitochondrial membrane potential. (**C**) Immunofluorescence images of mitochondrial superoxide content using MitoSoX. Data are represented as the mean percentage ± SEM (*n*=3); ****P<0.0001, ***P<0.001, **P<0.01, *P<0.05; ns, non-significant.

Studies reported that hyperpolarization of mitochondrial membrane potential is associated with ferroptosis [53]. JC1 staining of mitochondria revealed a reduction in mitochondrial membrane depolarization in MLT-treated groups compared with FFA treatment (Figure 5A,B) indicating a positive change by MLT. To identify mitochondrial ROS, particularly superoxide, mitoSOX-based assays are frequently utilized [54]. A decrease in mitochondrial ROS was observed under confocal microscopy among MLT treated groups in a dose-dependent manner compared with FFA, when administered with mitoSOX Red (Figure 5C).

### Melatonin inhibited metabolic markers associated with lipogenesis *in vitr*o

**Figure 6:**
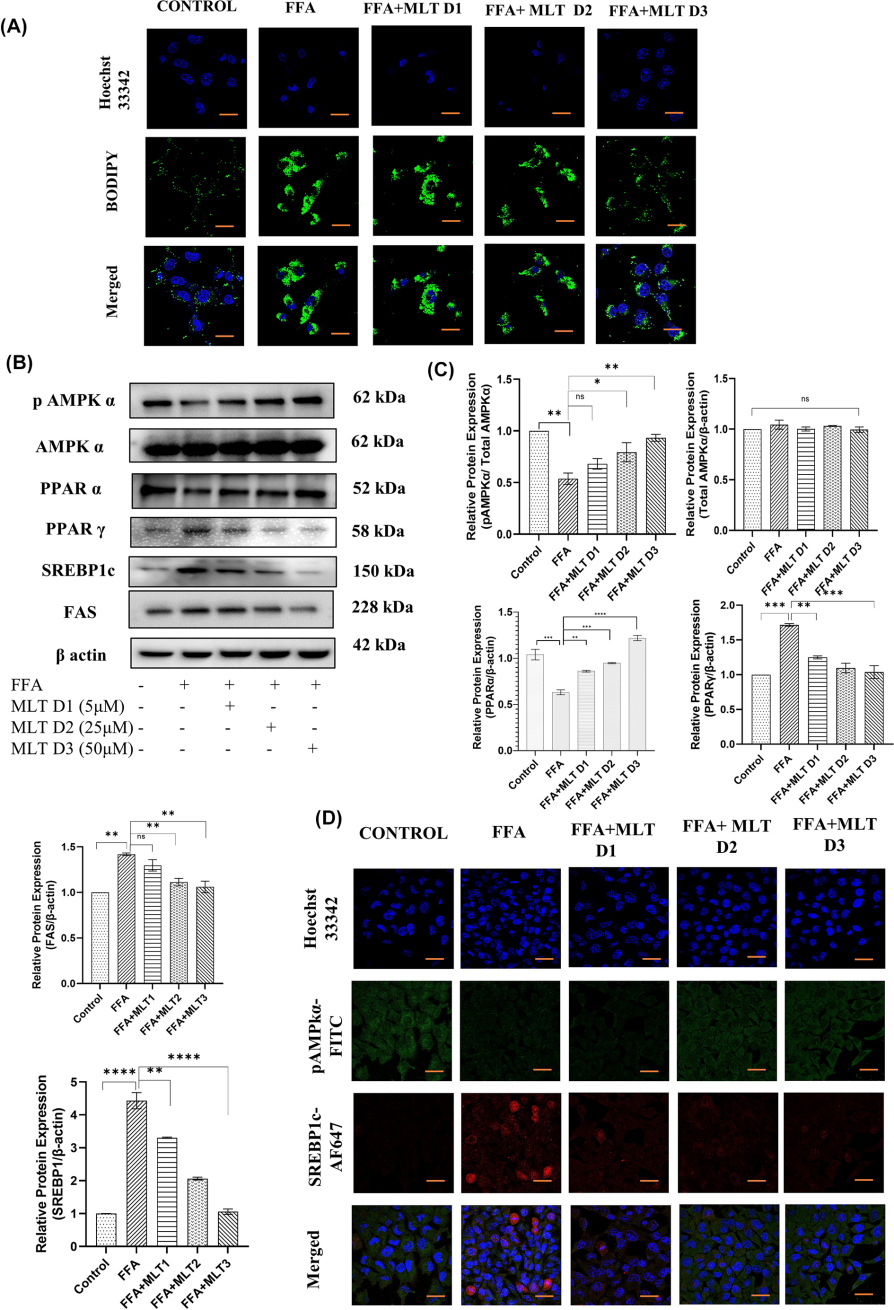
Melatonin inhibited metabolic markers associated with lipogenesis *in vitro* Analysis of lipid ROS and potential lipogenic markers. (**A**) BODIPY staining of HepG2 cells treated with FFA and different doses of MLT. (**B**) Western blot analyses of pAMPKα, total AMPKα, PPARα, PPARγ, SREBP1c and FAS are shown. (**C**) Bar graphs show the quantification of total AMPKα/β-Actin, pAMPKα/Total AMPKα, PPARα/β-Actin, PPARγ/β-Actin, FAS/β-Actin and SREBP1c/β-Actin, respectively. (**D**) Immunofluorescence images of expression of pAMPKα and SREBP1c; scale bar: 50 µm. Data are represented as the mean percentage ± SEM (*n*=3); ****P<0.0001, ***P<0.001, **P<0.01, *P<0.05; ns, non-significant.

Lipid accumulation and subsequent peroxides generation are important events leading to ferroptosis [55]. Factors affecting anabolic processes like lipogenesis are therefore crucial for the assessment of ferroptosis. In the present study, BODIPY staining of HepG2 cells exposed the capability of MLT in reversal of the process of lipid deposition. Formation of lipid droplets had been drastically reduced with increasing dose of MLT (FFA+MLT D1, FFA+MLT D2 and FFA+MLT D3) compared with the cells treated only with FFA (Figure 6A). SREBP1c is an exclusive lipogenic transcription factor responsible for transcribing genes like FAS, gene responsible for the formation of fatty acid synthase, which further regulates triglycerides formation and subsequent ROS generation. pAMPK acts as an inhibitor of SREBP1c [56]. Studies also reported that PPARα and PPARγ together regulates lipid homeostasis [57]. Western blot analysis of key regulatory proteins related to lipogenesis and subsequent events reflects gradual decrease in expression of PPARγ, SREBP1c and FAS and gradual increase in expression of pAMPK and PPARα with increasing dose of MLT compared with only FFA treatment (Figure 6B,C). This suggests ameliorative property of MLT against lipogenesis. Confocal microscopy of the expression of pAMPKα and SREBP1c also supports the results of immunoblotting (Figure 6D). Therefore, MLT can be concluded as an effective modulator of lipogenesis and associated events.

### Melatonin improved physiological parameters in C57BL/6 mice

**Figure 7:**
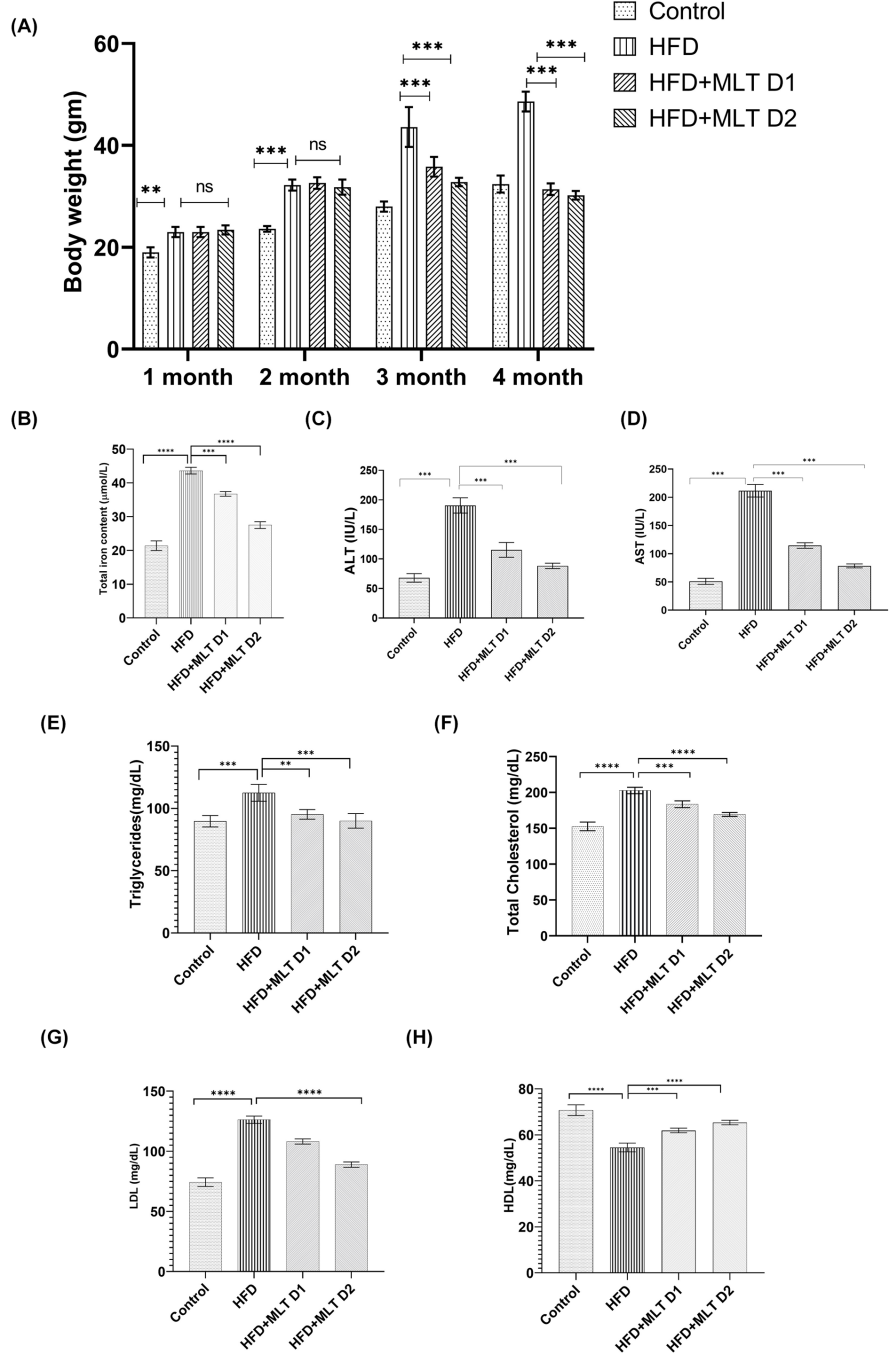
Melatonin improved physiological parameters in C57BL/6 mice Changes in physiological parameters in HFD-fed C57BL/6 mice with/without MLT treatment. (**A**) Body weight changes of mice in different time intervals. (**B**) Intracellular iron content of Liver tissue lysates. (**C,D**) ALT and AST level. (**E–H**) Serum level of triglycerides, total cholesterol, LDL and HDL. Data are represented as the mean percentage ± SEM (*n*=3); ****P<0.0001, ***P<0.001, **P<0.01, *P<0.05; ns, non-significant.

MLT showed its promising effect in C57BL/6 mice more or less similar to *in vitro* studies. Mice treated with MLT showed a decreasing pattern of their body weight (Figure 7A). Since iron deposition is key to development of ferroptosis, intracellular iron content of liver tissues of C57BL/6 mice was measured and results showed decreased intracellular iron content in mice administered with higher dose of MLT (HFD+MLT D1) compared with control and HFD treatment (Figure 7B). Comparable changes in ALT and AST level (Figure 7C,D) were also noted with increasing doses of MLT compared with HFD treated mice. Similarly, decrease in cholesterol, triglycerides and LDL level and increase in HDL levels were also observed following MLT administration (Figure 7E–H). This alteration overall suggests the role of MLT toward amelioration of body weight and overall liver health.

### Melatonin augmented recovery from NAFLD in C57BL/6 mice

**Figure 8:**
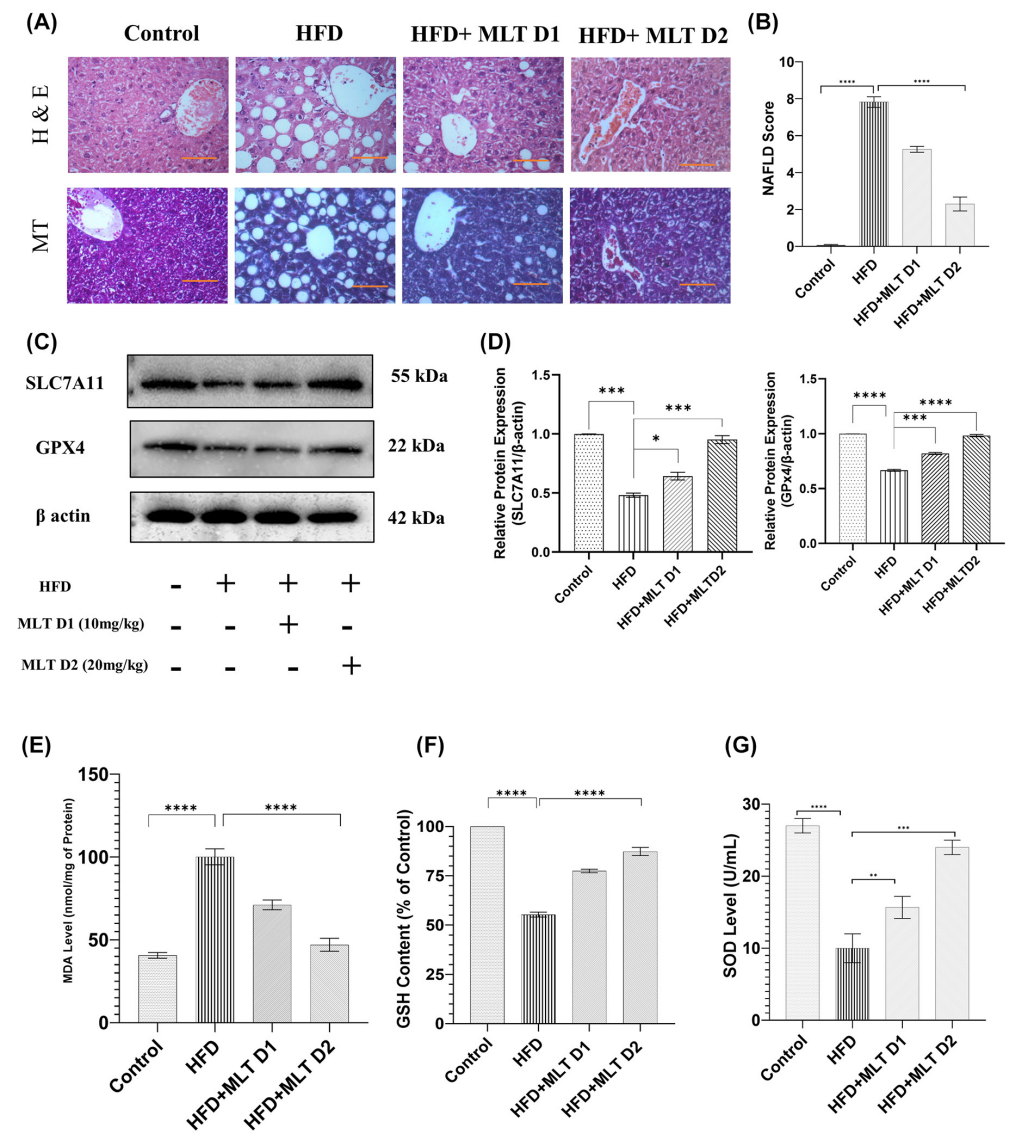
Melatonin augmented recovery from NAFLD in C57BL/6 mice Modulation of NAFLD-mediated histopathological and biochemical changes. (**A**) H&E and MT staining of HFD and MLT treated mice groups; scale bar = 40 µm. (**B**) Bar graph shows the NAFLD score. (**C**) Western blot analysis of SLC7A11 and GPX4. (**D**) Bar graphs show the quantification of SLC7A11/β-Actin and GPX4/β-Actin respectively. (**E–G**) Bar graph represents MDA level, GSH content and SOD level analyzed using Tissue lysates. Data are represented as the mean percentage ± SEM (*n*=3); ****P<0.0001, ***P<0.001, **P<0.01, *P<0.05; ns, non-significant.

**Figure 9:**
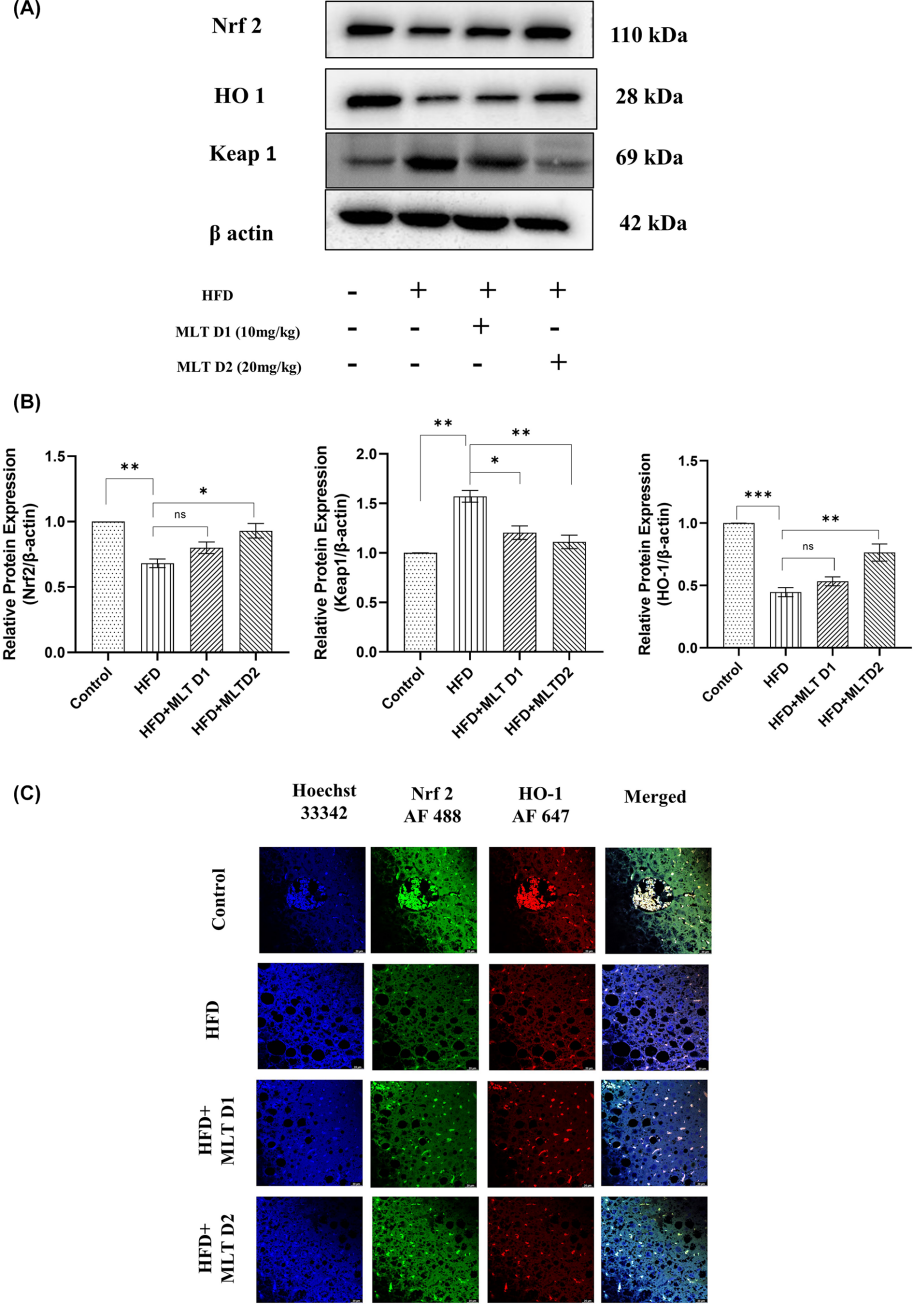
Melatonin augmented recovery from NAFLD in C57BL/6 mice Alteration of key protein markers of antioxidant gene expression. (**A**) Western blot analysis of Nrf2, HO-1 and Keap-1 in HFD- and MLT-treated mice groups. (**B**) Bar graphs show the quantification of Nrf2/β-Actin, HO-1/β-Actin and Keap-1/β-Actin, respectively. (**C**) Immunofluorescence images of expression of Nrf2 and HO-1; scale bar = 20 µm. Data are represented as the mean percentage ± SEM (*n*=3); ****P<0.0001, ***P<0.001, **P<0.01, *P<0.05; ns, non-significant.

Background of histopathological architecture was carefully examined to assess the grade of damage. HFD-fed liver showed an extensive histopathological change including ballooning degeneration of hepatocytes, Mallory’s hyaline and lobular inflammation. A reduction of ballooning degeneration was noted upon treatment with lower dose (D1) of MLT and a significant reduction was seen on higher dose (D2) administration in HFD fed mice indicating the inhibition of lipid droplet accumulation at higher dose. Mice with controlled diet was in stage-0 NAFLD, while, mice fed with HFD diet showed stage-3 of NAFLD (characterized by lobular inflammation, degeneration of hepatocytes along with ballooning, macro-vesicular steatosis, Mallory’s hyaline and fibrosis), HFD+MLT D1 mice had stage-2 (characterized by hepatocyte ballooning, micro-vesicular steatosis along with lobular inflammation), and HFD+ MLT D2 mice had stage-1 (characterized by simple steatosis). As per the cumulative damage scoring, HFD fed group was classified as grade 3 (severe), HFD+MLT D1 group as grade 2 (moderate) and HFD+MLT D2 group as stage 1 (mild) (Figure 8A,B). MLT treatment in mice also increased the level of SLC7A11 and GPX4, two crucial markers of ferroptosis signifying a possible recovery from ferroptosis induction (Figure 8C,D). MLT decreased lipid peroxidation, as implied by reduction in MDA level, similar to *in vitro* studies (Figure 8E). Significant increase in antioxidant level (GSH and SOD) was seen in MLT-treated mice groups supporting the re-establishment of redox homeostasis (Figure 8F,G). Nrf2 and HO-1, the key modulators of labile iron pool, displayed increased expression, respectively, in MLT-treated group in Western blot analysis (Figure 9A,B), which was further validated by observation of their similar expression pattern in confocal microscopy (Figure 9C).

### *In vivo* lipogenic markers altered following melatonin administration

**Figure 10:**
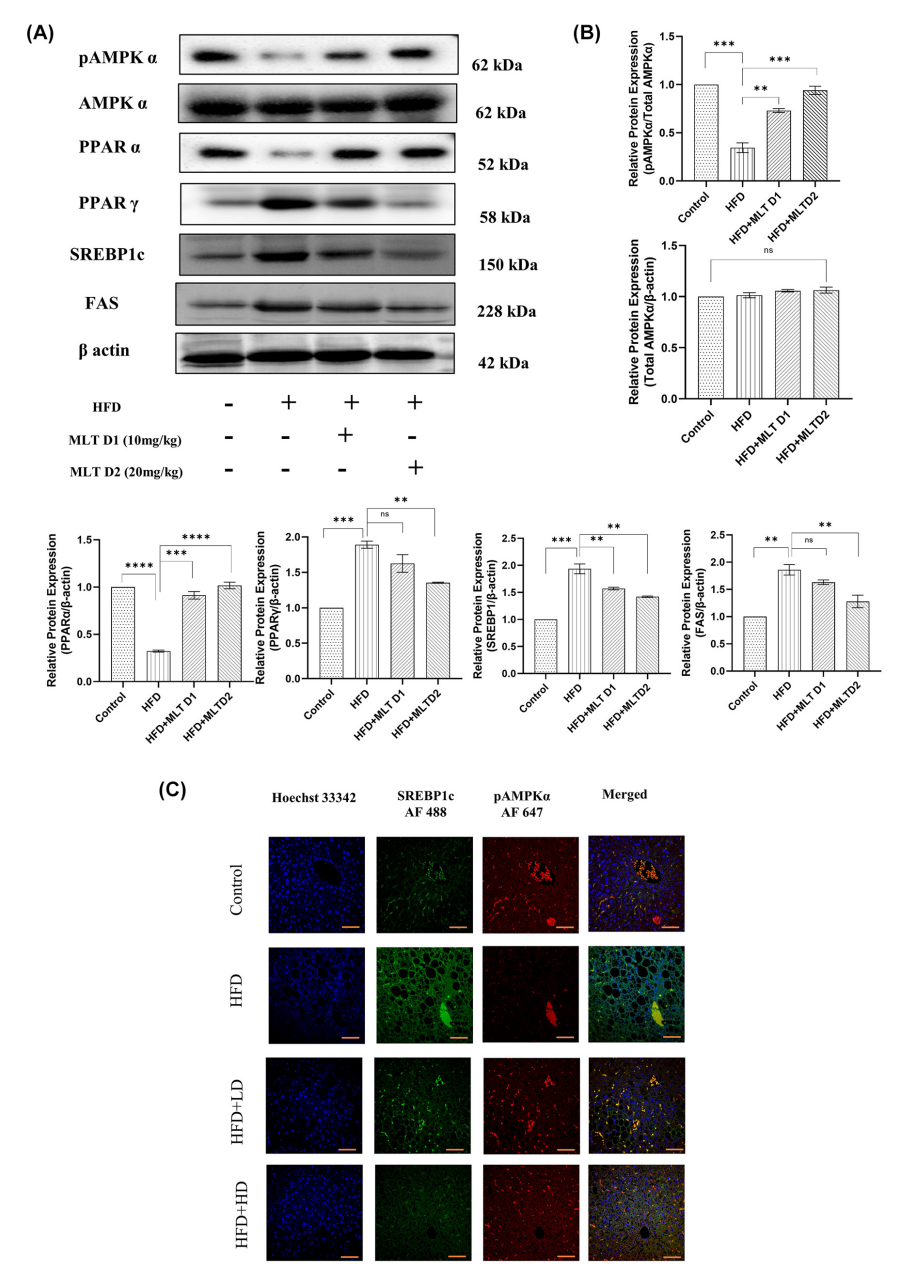
*In vivo* lipogenic markers altered following melatonin administration Melatonin re-established lipogenic protein levels. (**A**) Western blot analysis of pAMPKα, Total AMPKα, PPARα, PPARγ, SREBP1c and FAS. (**B**) Bar graphs show the quantification of total AMPKα /β-Actin, pAMPKα/total AMPKα, PPARα/β-Actin, PPARγ/β-Actin, FAS/β-Actin, and SREBP1c/β-Actin, respectively. (**C**) Immunofluorescence images of expression of pAMPKα and SREBP1c; scale bar: 40 µm. Data are represented as the mean percentage ± SEM (*n*=3); ****P<0.0001, ***P<0.001, **P<0.01, *P<0.05; ns, non-significant.

Similar to cells, lipid peroxidation caused by lipid accumulation is the triggering factor for ferroptosis *in vivo*. Increased lipid accumulation causes lipogenesis leading to lipid ROS formation [58,59]. Dose-dependent administration of melatonin in HFD-fed C57BL/6 mice significantly modified expression levels of proteins involved in lipogenesis, exactly similar to the pattern as seen from *in vitro* studies. Western blot analysis of lipogenic proteins showed decrease in expression of SREBP1c, PPARγ, and FAS and increase in expression of pAMPK and PPARα with increasing doses of MLT compared with only HFD administration (Figure 10A,B). If not inhibited by pAMPK, SREBP1c has the potential to regulate the expression of lipogenic genes [60]. Hence, their expression pattern should be confirmed in more than one way. Due to this, confocal microscopy of these two proteins was performed to further confirm their expression pattern and the results revealed the same pattern as in Western blot analysis (Figure 10C).

## Discussion

NAFLD encompasses a spectrum of liver diseases ranging from relatively milder forms such as steatosis to the more severe forms such as NASH, advanced fibrosis, cirrhosis and liver failure. The chief characteristic of NAFLD is the accumulation of fat in liver cells [3]. Several studies indicated the involvement of ferroptosis in different diseases including cancer, neurodegeneration, heart disease, and in particular, liver diseases. Ferroptosis is a kind of cell death that differs ideologically from other types of cell deaths due to its dependence on intracellular iron deposition [61]. Enhanced lipid peroxidation due to excess iron accumulation plays a principal role in the development of ferroptosis [62]. However, since increased iron overload is an integral part of liver diseases [63]; hence, ferroptosis should hold a strong role in the development of NAFLD. Melatonin is the highly explored pineal hormone due to its antioxidant and anti-inflammatory properties. Studies also reported the role of melatonin in amelioration of ferroptosis in acute sleep deprivation-induced memory loss [64], osteoporosis [65], PM2.5-induced lung injury [66], etc. The objective of the present study was to explore its potential role in NAFLD induced hepatic ferroptosis. In order to achieve our goal, an NAFLD microenvironment was induced in HepG2 cells by using FFA [67], and the model was further validated using the mice model of NAFLD induced by HFD administration in C57BL/6 mice, as described by previous studies [68]. Hepatic lipogenesis and subsequent lipid peroxidation is an important factor contributing to NAFLD. Studies also reported that dysregulated iron metabolism, deposition of PUFA-PLs (polyunsaturated fatty acids phospholipids) and lipid peroxidation also leads to ferroptosis [69]. Since both NAFLD and ferroptosis are triggered by the same kind of events, there should be a link between these two. Ma et al. stated that high glucose-induced ferroptosis in Type 2 diabetic osteoporosis can be suppressed by melatonin [65]. However, no other studies have yet explored the protective role of MLT in NAFLD related ferroptosis. In our study, we primarily focused on the defensive role of MLT against ferroptosis in NAFLD. In order to do that, first Oil Red O staining was done to observe lipid accumulation in cell and subsequent lipid peroxidation was evaluated using MDA level. The results indicated a reduction in lipid content and MDA level following MLT administration in HepG2 cells suggesting the protective role of MLT against lipogenesis and lipid peroxidation. Numerous compounds like erastin, ferroptosis inducer 56 [FINF56] RAS-selective lethal [RSL3] and ferroptosis inducer endoperoxide [FINO2] caused loss of GPX4 activity and uncontrolled lipid peroxidation, leading to cell death [70,71]. To find out the exact mechanism through which MLT exerted its effect against ferroptosis in NAFLD, expression of two key proteins SLC7A11 and GPX4 were analyzed, whose dysregulation causes accumulation of ROS leading to enhanced lipid peroxidation and ferroptosis [72]. The results signified the success of MLT in increasing their level back to normal from FFA induced alteration. Normalization of intracellular iron content both in MLT-treated HepG2 cells and also in hepatocytes of C57BL/6 indicated a recovery from ferroptosis.

Previous studies indicated involvement of the Nrf2/HO-1 pathway in lipid peroxidation and ferroptosis [65]. Studies reported inhibition of Nrf2, withdraws the resistance of head and neck cancer to GPX4 inhibitor-induced ferroptosis [33]. This evidence confirms the Nrf2 mediated regulation of intracellular iron metabolism to regulate ferroptosis. To assess whether MLT modifies the Nrf2/HO-1 antioxidant axis in this model, Western blot analysis of Nrf2, Keap-1 and HO-1 proteins were executed and increased Nrf2 and HO-1 expression and consequently decreased expression of Keap-1 were found in MLT treated cells demonstrating that MLT helps translocation of Nrf2 to nucleus and transcription of antioxidant genes like HO-1. Besides this, treatment with Nrf2 SiRNA also concluded the role of Nrf2 in this regard. Therefore, it is evident that MLT enhanced the Nrf2/HO-1-mediated signaling to counteract ferroptosis.

Since SLC7A11 and GPX4 level reduced following FFA administration, dysregulation of these two can increase intracellular ROS, which may contribute to lipid peroxidation. Several studies reported the antioxidant effect of MLT [73] and the results from quantification of GSH and SOD also supported the fact, signifying replenishment of oxidative homeostasis by MLT both *in vivo* and *in vitro*. Lipid peroxidation results from imbalance between peroxide generation and its elimination, sometimes leading to iron deposition and oxidative form of cell death, ferroptosis [74]. Since, increased lipid accumulation increases ROS production resulting in lipid peroxidation [75], the present study also explored BODIPY staining of lipid ROS and analysis of the protein markers of lipogenic pathway through Western blot and confocal microscopy. BODIPY staining of both HepG2 cells and liver tissues of C57BL/6 mice revealed that melatonin performed well in reduction of lipid ROS both *in vivo* and *in vitro* due to its antioxidant activity. Studies reported that pAMPK plays an important role in controlling lipogenesis by modulating SREBP1c [56,76]. In the present study, increase in pAMPK expression upon MLT administration both in HepG2 cells and liver hepatocytes revealed that MLT had a control over pAMPK to regulate other lipogenic markers like SREBP1c, FAS. Conversely, MLT also decreased PPARγ and increased PPARα, the other regulators of lipogenesis.

In conclusion, MLT could counteract ferroptosis through inhibition of lipogenesis, reduction of ROS generation, elevation of SLC7A11 and GPX4 expression and modulation of Nrf2/HO-1 axis, strongly suggesting its therapeutic efficacy against ferroptosis.

## Conclusion

In summary, our results suggested the first evidences of Melatonin’s protective effect against ferroptosis in NAFLD. Although the involvement of Nrf2/HO-1 pathway is described in various studies as a defensive system against endogenous ROS, our study explored the role of melatonin in triggering Nrf2 to induce downstream antioxidant genes expression as well as melatonin’s control over SLC7A11 and GPX4 to modulate ROS generation and lipid peroxidation. The present study also revealed that melatonin even altered major lipogenic markers like pAMPKα, SREBP1c to hinder lipogenesis in first hand to inhibit further lipid ROS generation due lipid accumulation and subsequent ferroptosis.

## Clinical perspectives

- A handful of studies have been conducted to assess the potential of melatonin in different ferroptosis models. However, no study yet addressed the integral mechanism of Melatonin’s action in NAFLD-induced ferroptosis model.
- Study using both *in vitro* and *in vivo* model revealed that melatonin had a control over both cellular redox homeostasis and cellular signalling network as well as proteins associated with lipogenesis to combat ferroptosis.
- Our study was first of its kind that strategically explored the role of key Pineal hormone against ferroptosis. Therefore, melatonin can be further assessed preclinically in patients with NAFLD to alleviate the progression of the disease with minimized side effects due to its inherent presence.

## Supplementary Material

Supplementary Figures S1-S2 and Raw Western Blot Figures

## Data Availability

Data will be available upon request.

## Abbreviations

FFA: free fatty acid
HFD: high-fat diet
MDA: malondialdehyde
MLT: melatonin
PPAR: peroxisome proliferator-activated receptor
NAFLD: non-alcoholic fatty liver disease
NASH: non-alcoholic steatohepatitis
PAGE: polyacrylamide gel electrophoresis
ROS: reactive oxygen species
SAS: sulphasalazine
