# Supplementary material for: Melatonin targets ferroptosis through bimodal alteration of redox environment and cellular pathways in NAFLD model
Source: Biosci Rep. 2023 Oct 6;43(10):BSR20230128. doi: 10.1042/BSR20230128 (PMC10560965; doi:10.1042/BSR20230128)
Supplement: Supplementary Figures S1-S2 and Raw Western Blot Figures [file BSR-2023-0128_supp.pdf]

Supplementary Information:

Supplementary Figure 1 : Establishment of In-vivo and in- vitro model and MLT treatment design.

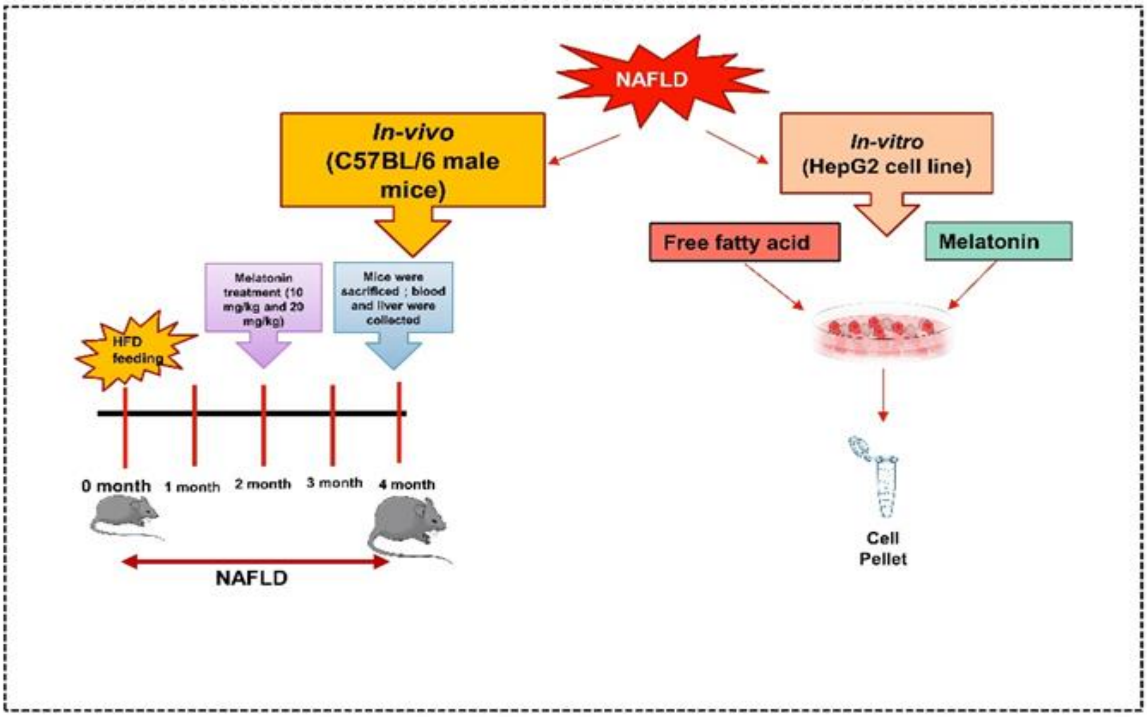

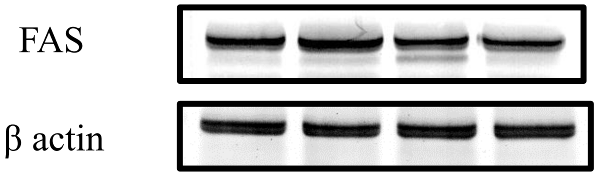

**FFA**	-	+	+	+
**MLT D3**	-	-	+	-
**FAS Inhibitor**	-	-	-	+

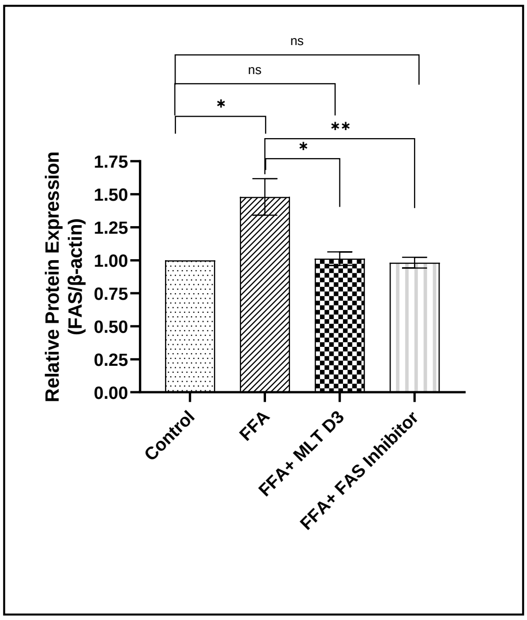

Supplementary Figure 2: Bar Diagram representing relative viability of HepG2 cells upon Melatonin treatment.

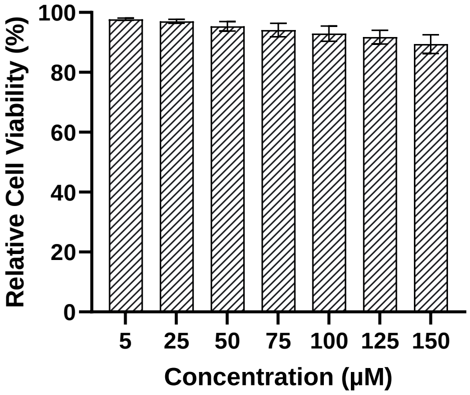

Full western Blot images of "Supplementary Figure 1"

Raw Western Blot Images:

Figure 1

(D)

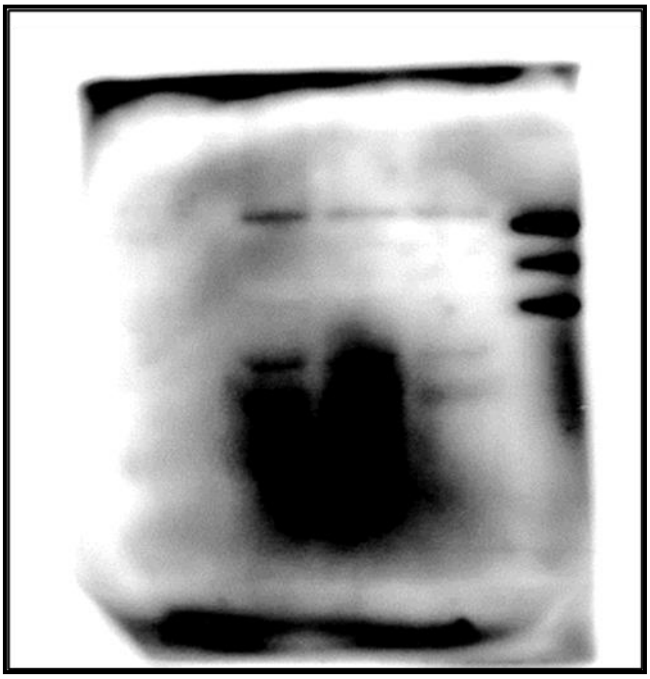

SLC7A11

55kDa

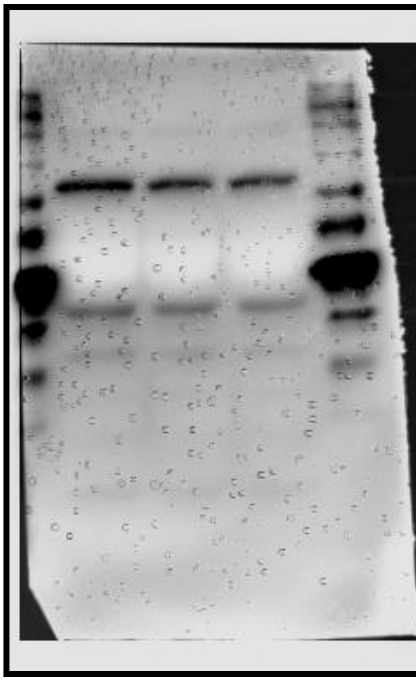

GPX4

22kDa

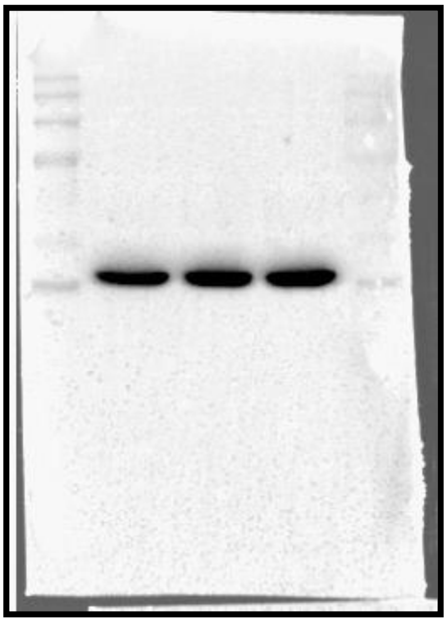

β actin

42kDa

Figure 2

(C)

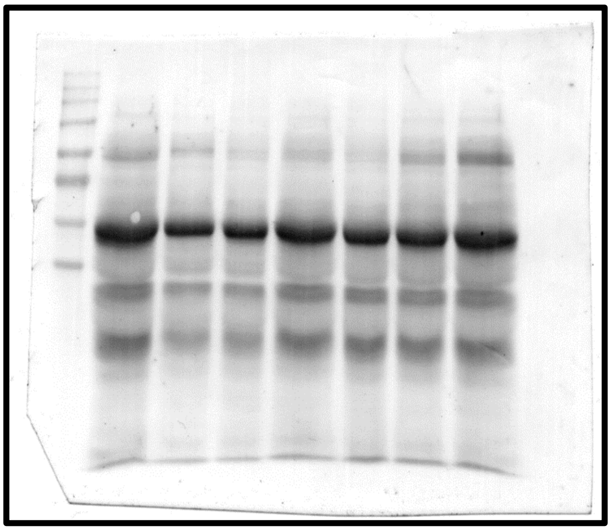

SLC7A11
55kDa

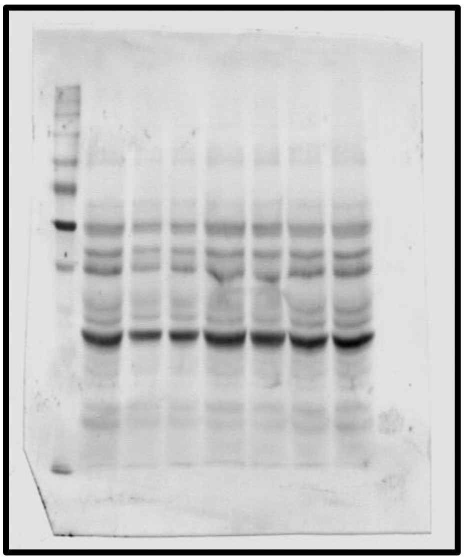

GPX4

22 kDa

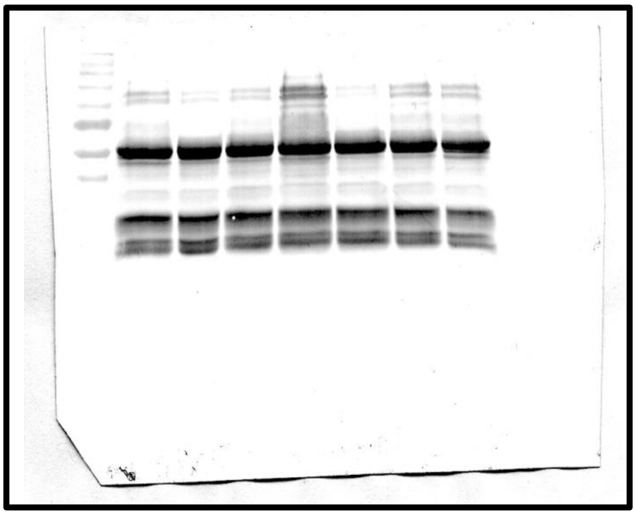

β actin

42 kDa

Figure 3

(F)

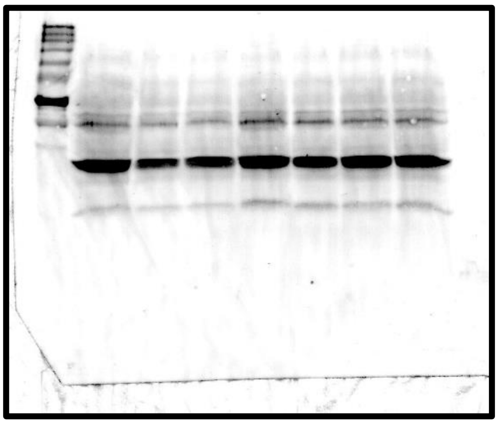

HO - 1

28 kDa

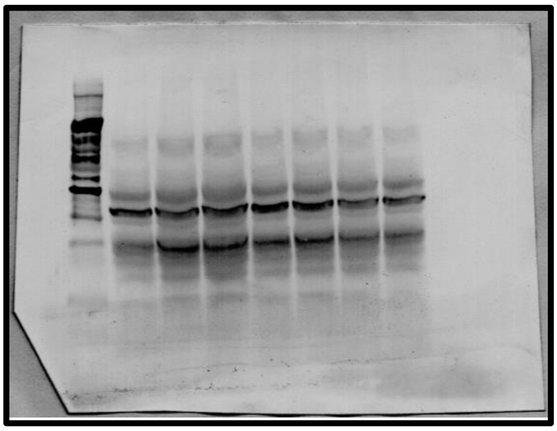

Keap 1

69 kDa

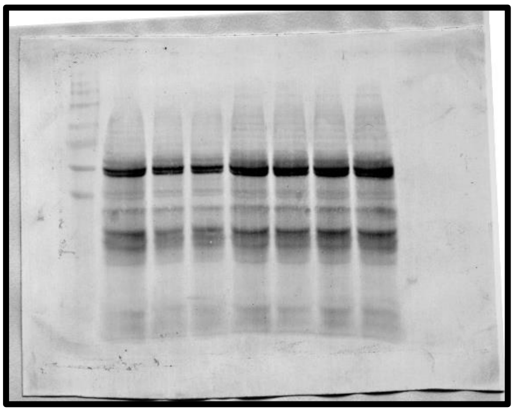

Nrf2

110 kDa

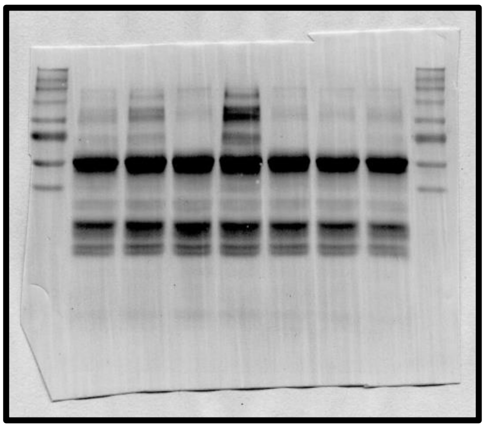

β actin

42 kDa

Figure 4

(A)

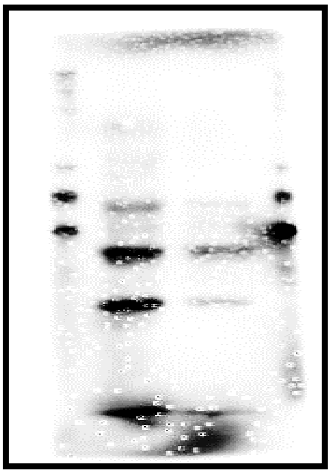

Nrf2

110 kDa

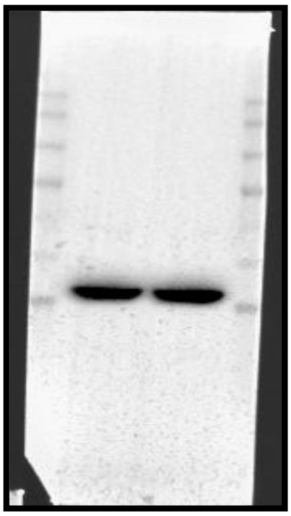

β actin

42 kDa

(C)

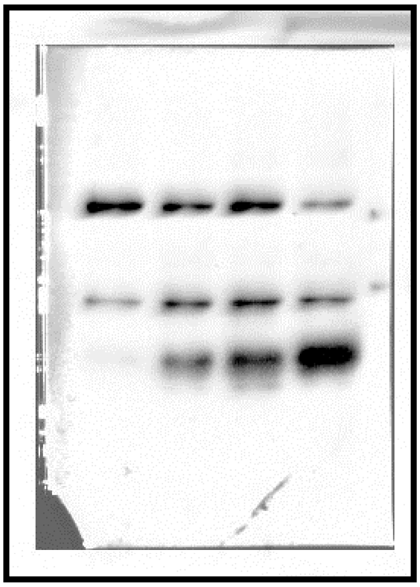

SLC7A11

55 kDa

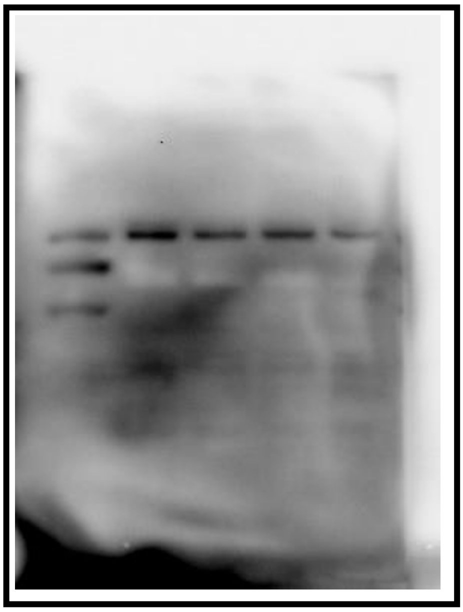

GPX4

22 kDa

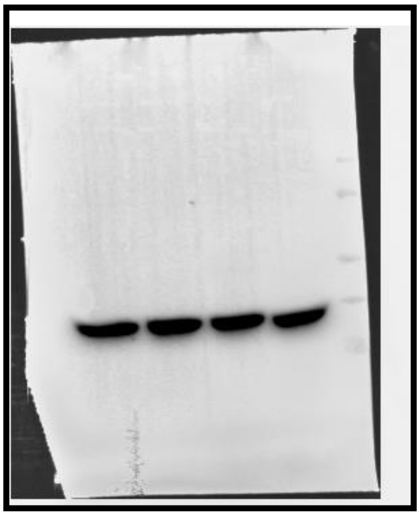

42 kDa

Figure 6

(B)

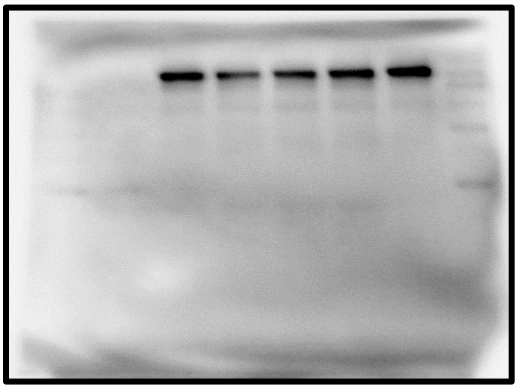

p AMPK α

62 kDa

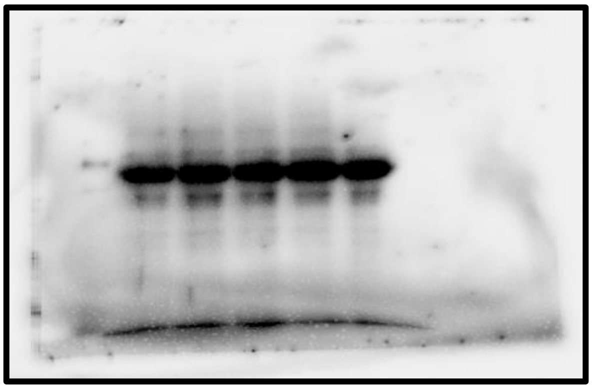

AMPK α

62 kDa

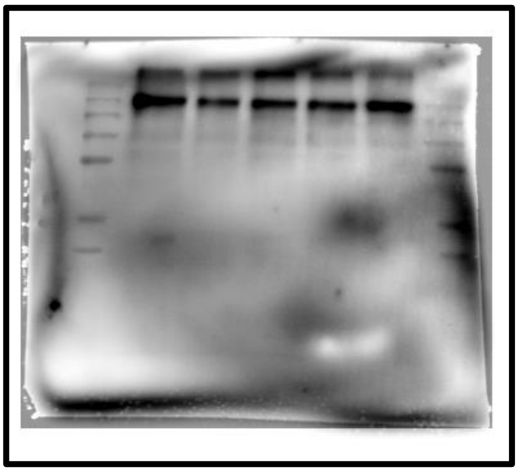

PPAR α

52 kDa

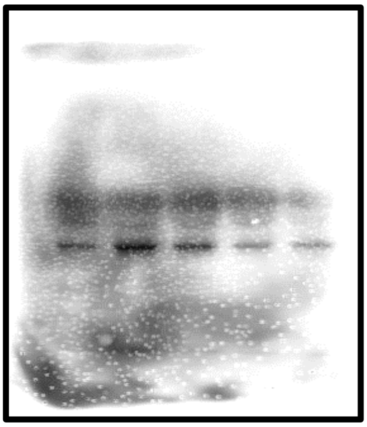

PPAR γ

58kDa

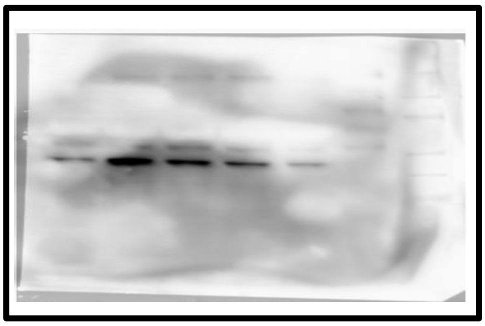

SREBP1c

150kDa

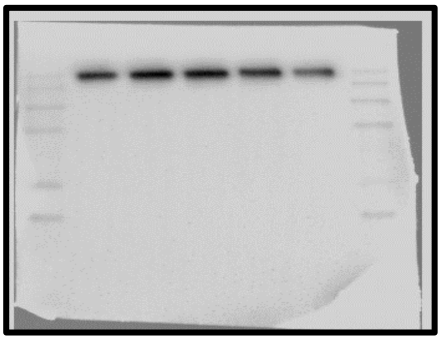

FAS

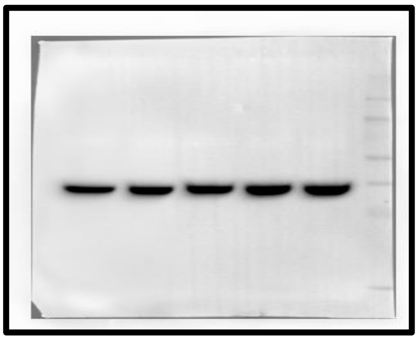

β actin

42 kDa

228kDa

Figure : 8

(C)

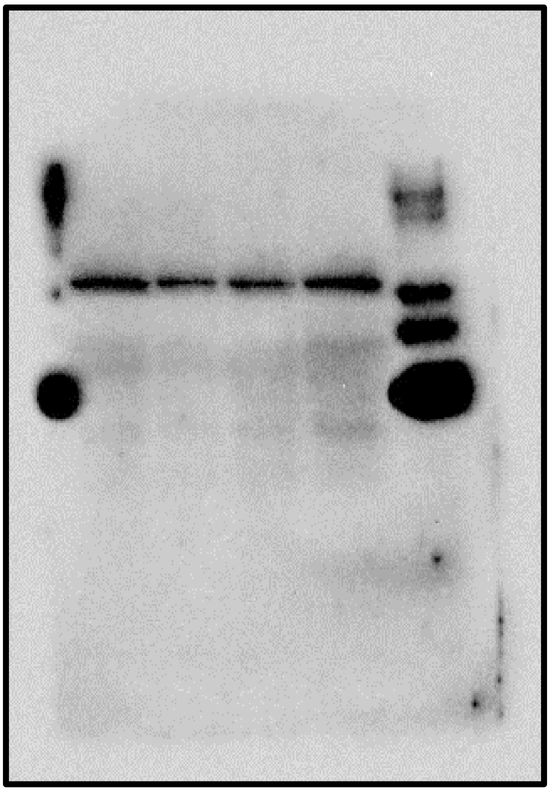

SLC7A11

55 kDa

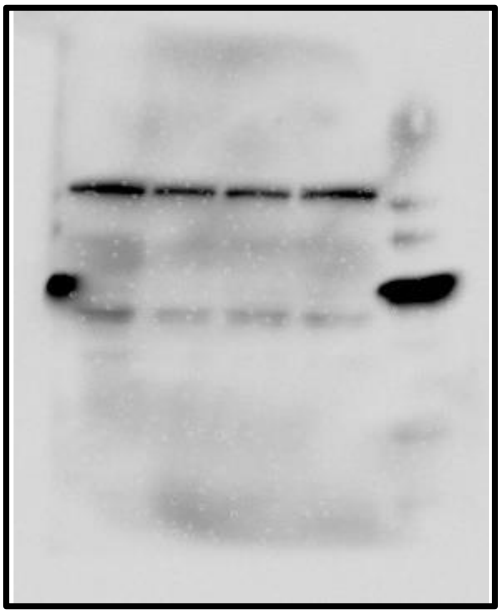

GPX4

22 kDa

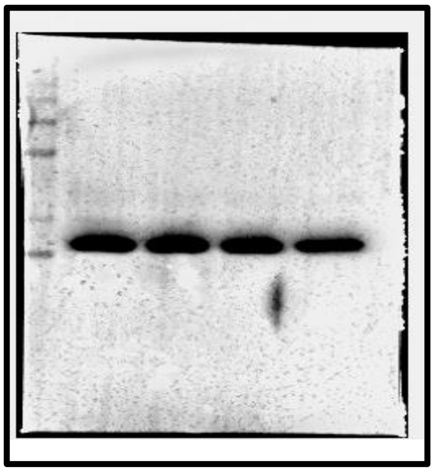

β actin

42 kDa

Figure: 9

(A)

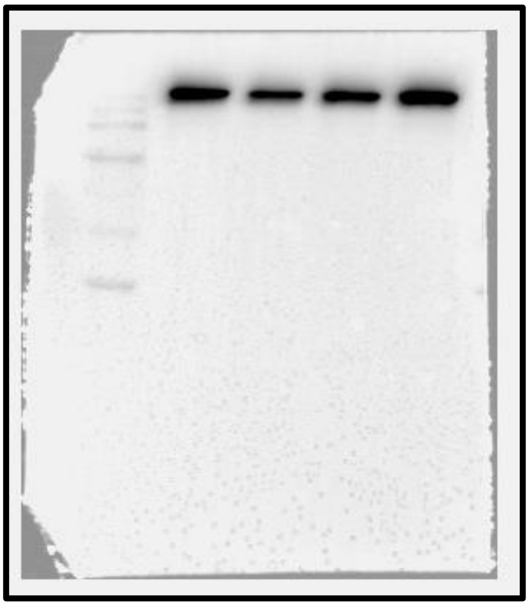

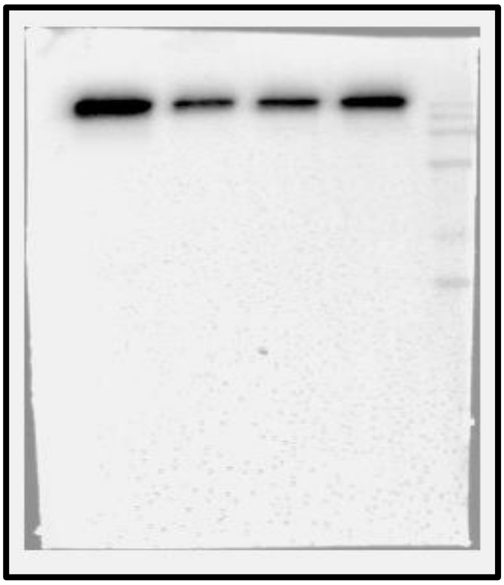

Nrf 2
110 kDa

HO 1
28 kDa

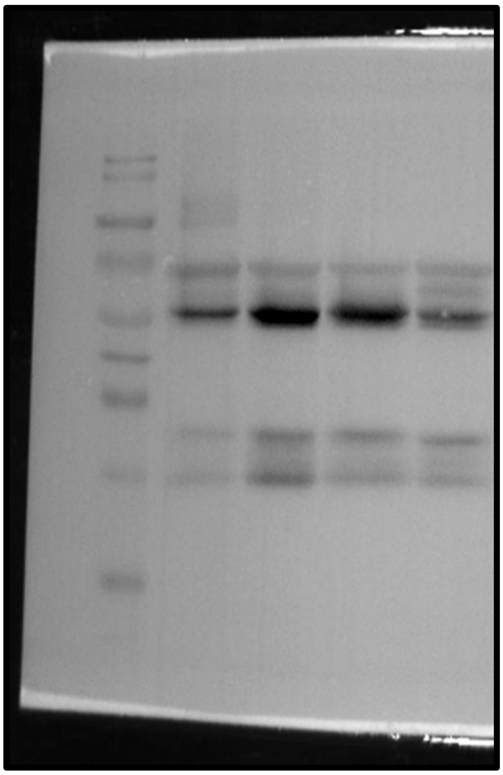

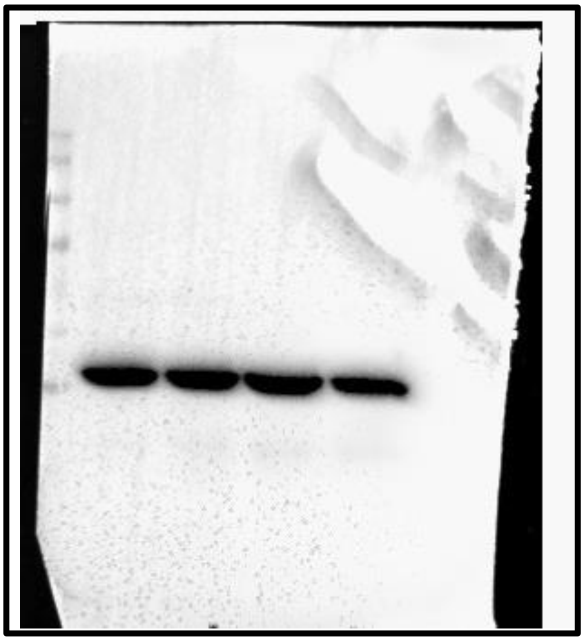

Figure : 10

(A)

Keap 1

69 kDa

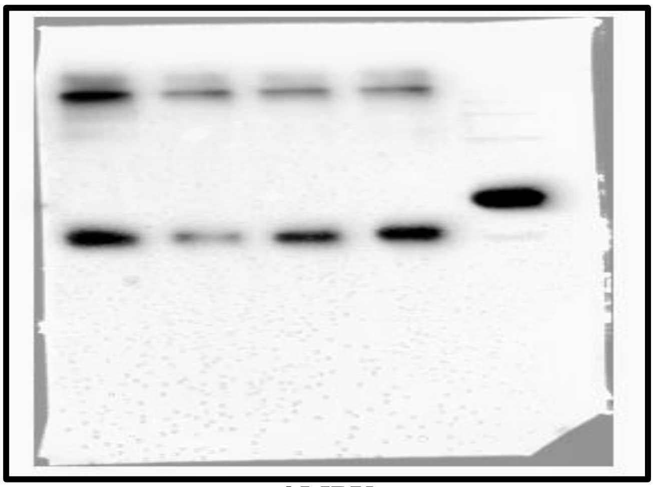

β actin

42 kDa

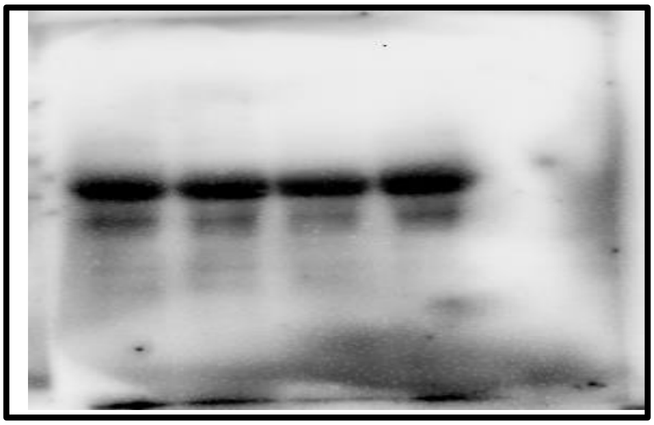

AMPK α

62 kDa

pAMPKα

62 kDa

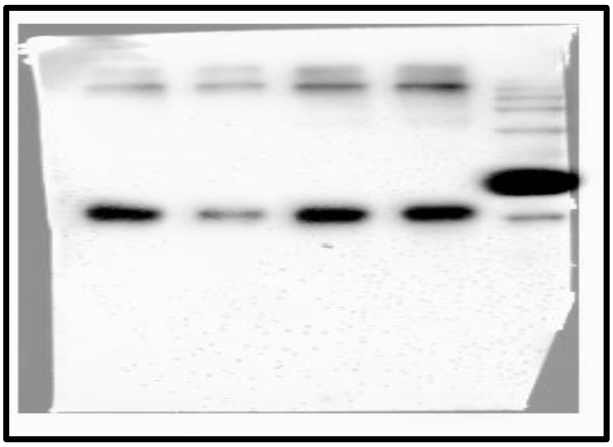

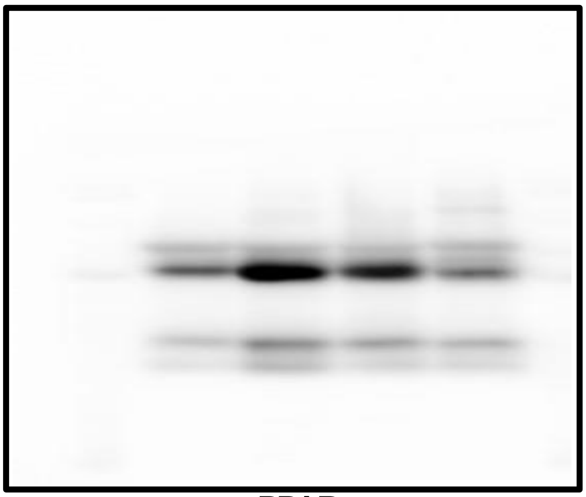

PPAR α

52 kDa

PPAR γ

58 kDa

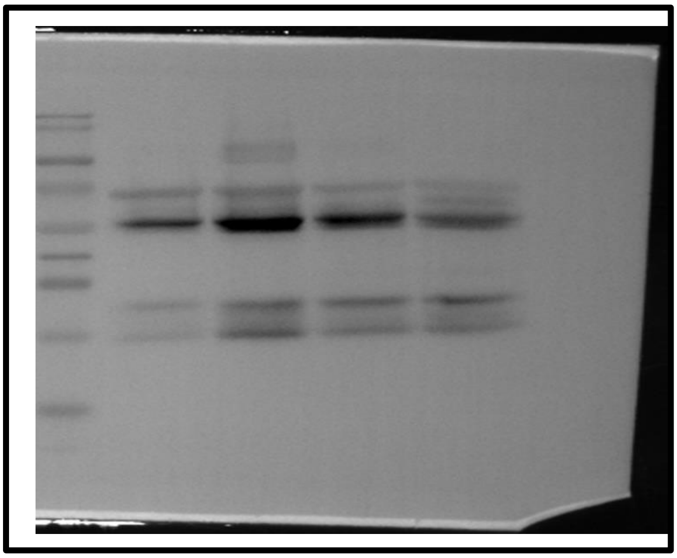

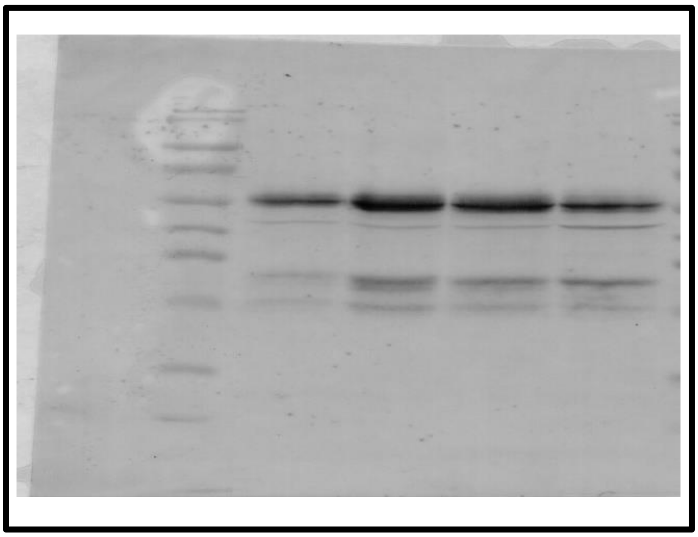

SREBP1c

150 kDa

FAS

228 kDa

Figure : 10

(A)

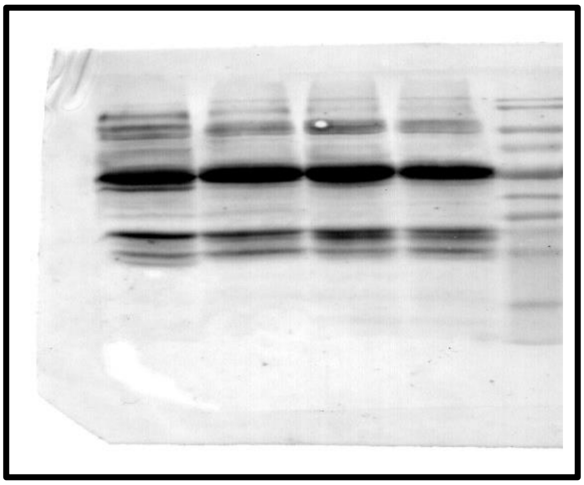

β actin

42 kDa

Supplementary Figure 1:

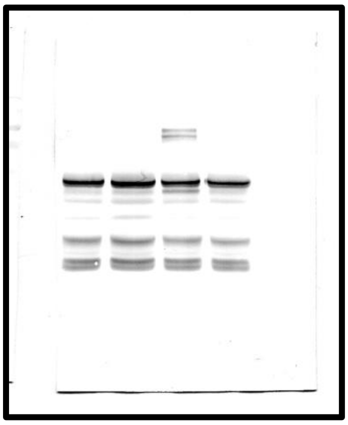

FAS

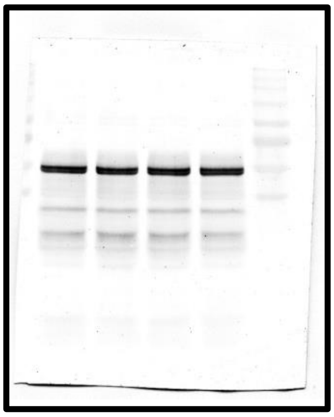

β actin
